# Conditioning the soil microbiome through plant–soil feedbacks suppresses an aboveground insect pest

**DOI:** 10.1111/nph.16385

**Published:** 2020-02-03

**Authors:** Ana Pineda, Ian Kaplan, S. Emilia Hannula, Wadih Ghanem, T. Martijn Bezemer

**Affiliations:** ^1^ Department of Terrestrial Ecology Netherlands Institute of Ecology (NIOO‐KNAW) Wageningen 6700 AB the Netherlands; ^2^ Department of Entomology Purdue University West Lafayette IN 47907 USA; ^3^ Institute of Biology Section Plant Ecology and Phytochemistry Leiden University Leiden 2300 RA the Netherlands

**Keywords:** below-aboveground, herbivores, microbe–plant–insect interactions, microbiome‐induced systemic resistance, plant–soil–insect feedbacks, soil microbiomes, sustainability, thrips

## Abstract

- Soils and their microbiomes are now recognized as key components of plant health, but how to steer those microbiomes to obtain their beneficial functions is still unknown. Here, we assess whether plant–soil feedbacks can be applied in a crop system to shape soil microbiomes that suppress herbivorous insects in above‐ground tissues.
- We used four grass and four forb species to condition living soil. Then we inoculated those soil microbiomes into sterilized soil and grew chrysanthemum as a focal plant. We evaluated the soil microbiome in the inocula and after chrysanthemum growth, as well as plant and herbivore parameters.
- We show that inocula and inoculated soil in which a focal plant had grown harbor remarkably different microbiomes, with the focal plant exerting a strong negative effect on fungi, especially arbuscular mycorrhizal fungi. Soil inoculation consistently induced resistance against the thrips *Frankliniella occidentalis,* but not against the mite *Tetranychus urticae,* when compared with sterilized soil. Additionally, plant species shaped distinct microbiomes that had different effects on thrips, chlorogenic acid concentrations in leaves and plant growth.
- This study provides a proof‐of‐concept that the plant–soil feedback concept can be applied to steer soil microbiomes with the goal of inducing resistance above ground against herbivorous insects.

## Introduction

Soils are crucial for terrestrial life (Wall *et al*., 2015). The soil is where most terrestrial plants start their growth and, more than a simple substrate, is home to a diverse community of microbes. Several of these microbes provide plants with key functions, such as enhanced growth via improved nutrition or suppression of soil pathogens (Pieterse *et al*., 2016; Raaijmakers & Mazzola, 2016). An important soil service is the protection of above‐ground plant tissues against pests and diseases (Bardgett & Wardle, 2010), and this could be used to improve sustainability in agriculture (Kaplan *et al*., 2018; Mariotte *et al*., 2018). The soil is the source of most beneficial microbes that colonize the rhizosphere (Bulgarelli *et al*., 2013) – the thin interface of root surface with attached soil – which are key players in plant immunity and overall plant performance. To date, soil management typically focuses on avoiding detrimental effects such as allelopathy or accumulation of pathogens and pests via crop rotation (Peralta *et al*., 2018). However, an exciting possibility is to manage soils to steer microbial communities to a desired beneficial state with a focus on promoting the presence and activity of beneficial microbes, instead of simply avoiding the pathogenic ones. Empirical evidence that this type of soil management can increase resistance in crops against above‐ground herbivores is, however, still lacking (Pineda *et al*., 2017).

It is well established that rhizosphere colonization by beneficial soil microbes can reduce the negative impact of above‐ground herbivores on plant growth (Pieterse *et al*., 2014; Pineda *et al*., 2017; Rashid & Chung, 2017). For example, soil microbes can prime plants to respond faster or stronger to their attackers, especially to cell‐feeding and leaf‐chewing herbivores (Martínez‐Medina *et al*., 2016). Until recently, research on microbe–plant–insect interactions has focused on the effects of a limited number of individual microbial strains, which often generate inconsistent results when applied in the field (Gadhave *et al*., 2016; Timmusk *et al*., 2017). An alternative approach is to focus on the complete microbiome. Several authors have argued that the introduction of more complex soil communities, rather than single species/strains, is necessary to achieve consistent enhancement of crop protection (Busby *et al*., 2017; Pineda *et al*., 2017), but so far, evidence of resistance against herbivores triggered either by such microbiome or by a single microbial strain functioning in a complex microbial community is scarce. Interestingly, studies with Arabidopsis or other wild plant species have shown, using soil sieving or sterilization, that the soil microbiome as a whole plays a significant role in inducing plant resistance to leaf‐feeding herbivorous insects (Badri *et al*., 2013; Hubbard *et al*., 2019; Wang *et al*., 2019). Until now, most studies on soil microbiomes have focused on building synthetic communities based on culturable organisms (Santhanam *et al*., 2015; Herrera Paredes *et al*., 2018) and the challenge is now how to manipulate those microbiomes to optimize induced resistance in crops.

One approach to steer soil microbiomes is to apply the ecological concept of plant–soil feedbacks (van der Putten *et al*., 2013; Bennett & Klironomos, 2018). Plants release primary and secondary metabolites through their roots that shape the soil and rhizosphere microbiome in a species‐specific way (Hu *et al*., 2018; Yuan *et al*., 2018). Thus, when a new plant grows in soil in which another plant had previously grown, its performance can be enhanced or reduced, depending on changes in the soil triggered by the first plant. Plant–soil feedbacks are well studied in the context of succession and invasion ecology to explain how different plant species interact (van der Putten *et al*., 2013). Notably, several recent studies indicate that plant–soil feedbacks also affect above‐ground plant–insect interactions in wild plant species (Kos *et al*., 2015; Heinen *et al*., 2018; Hannula *et al*., 2019). An important question is whether and how plant–soil feedbacks can be used to steer soil microbiomes to increase resistance of later‐growing crops to above‐ground pests (Kaplan *et al*., 2018; Mariotte *et al*., 2018).

Soil inoculation has been mainly studied in the context of restoration ecology, showing that degraded soils and their ecosystem functions can be restored quicker when soil microbiomes are inoculated (Middleton & Bever, 2012; Wubs *et al*., 2016). Agricultural fields and especially glasshouse soils are highly degraded, but the application of soil inoculation to restore the above‐ground functions of these soils is a new research field. Recently, we showed how inoculation with soils in which grasses had previously grown generally increased growth of chrysanthemum in the presence of the soil pathogen *Pythium* (Ma *et al*., 2017). This study supports the idea of applying the concept of plant–soil feedbacks to enhance the soil suppressiveness against pathogens (Schlatter *et al*., 2017). An important question that now needs to be answered is which part of the soil microbiome is altered by the first plant, and the extent to which these changes relate to the growth of the second plant. Plant–soil feedbacks are not static, and although largely overlooked in the plant–soil feedback literature, the second plant that grows in the soil will also influence the soil microbiome. Owing to the dynamic composition of the soil microbiome, if the most recent plant overrides the legacy of the first plant, inoculation with specific microbiomes may only influence the early growth phase of the second plant. However, two recent studies with a wild plant species show that plant‐specific legacy effects may persist even after another plant has grown in the soil (Bezemer *et al*., 2018; Wubs & Bezemer, 2018). If this is true, a single inoculation may influence plant growth for a much longer time, and this could be particularly important in crops that are grown repeatedly in the same soil.

The main goal of this study was to assess how inoculation with soil from grass and forb species alters soil microbiomes after growth of a second, focal plant, and whether soil inoculation alters resistance to above‐ground herbivorous pests in a crop system. Based on a previous screening of the effectiveness of soil inoculation with 37 wild plant species (Ma *et al*., 2017), we selected eight species (four grasses and four forbs) that previously improved the performance of chrysanthemum, to generate soil inocula with distinct microbiomes. We then inoculated sterilized soil with these species‐specific soils to assess chrysanthemum growth and we determined the composition of the soil microbiome in the inocula and in soil of chrysanthemum after this plant had been grown in the inoculated soils. Finally, we determined how inoculation influenced the resistance of chrysanthemum to two species of above‐ground herbivorous pests, thrips and mites. These are cell‐feeding herbivores and we hypothesized that inoculation would increase resistance against these pests via positive plant–microbe interactions. To understand phytochemical mechanisms underlying induced resistance effects, we measured foliar concentrations of chlorogenic acid, which is known to confer resistance to thrips (Leiss *et al*., 2009), as well as to increase after the plant interaction with beneficial microbes (Sanchez‐Bel *et al*., 2016).

Specifically, we asked: does inoculation with soil from different plant species and functional groups lead to different microbiomes in the soil after the same crop has grown in all inoculated soils; does soil inoculation alter plant growth and induce resistance against herbivorous pests; and which soil microbial groups in the inoculum and the crop microbiome correlate with plant growth and resistance to above‐ground herbivores?

## Materials and Methods

### Plants and herbivores

The focal plant in our study is *Dendranthema × grandiflora* (Ramat.) Kitam. cv Amadea (chrysanthemum, syn. *Chrysanthemum × morifolium* (Ramat.) Hemsl., Asteraceae). Chrysanthemum cuttings were provided by the breeding company Deliflor (Maasdijk, the Netherlands). Chrysanthemum is one of the major cut flower crops worldwide and is commonly cultivated in soil in glasshouses, which is sterilized regularly (after three to five growth cycles, roughly once per year) to control soil pathogens (Li *et al*., 2017). A culture of the thrips *Frankliniella occidentalis* was established on pods of Romano beans (*Vicia faba*) with a starting colony provided by the company Hazera Seeds (Made, the Netherlands). A culture of the spider mite *Tetranychus urticae* (line Sandpoort‐2) was established (Liu *et al*., 2017) with a starting colony kindly provided by the group of M. Kant (University of Amsterdam), and these were reared on detached leaves of Lima bean plants (*Phaseolus vulgaris* cv Speedy). More details are provided in the Supporting Information Methods S1.

To create different soil inocula, we selected eight wild plant species that are typical of natural grasslands in the Netherlands based on previous work where they exhibited positive plant–soil feedback effects on chrysanthemum growth (Ma *et al*., 2017). The species belong to two different functional groups: grasses (*Holcus lanatus* (HL), *Lolium perenne* (LP), *Alopecurus pratensis* (AP), *Festuca ovina* (FO)) and forbs (*Achillea millefolium* (AM), *Tripleurospermum maritimum* (TM), *Rumex acetosella* (RA), *Galium mollugo* (GM)).

### Soil conditioning, inoculation and plant growth

Two experiments were conducted in the experimental glasshouse facility at the NIOO‐KNAW (Wageningen, the Netherlands). Both experiments had two phases (see further details in Methods S2) a common procedure in plant–soil feedback studies. In the first phase, the conditioning phase, we grew over 2 months the eight plant species in monocultures in a living soil collected from a natural grassland to create species‐specific soil inocula. In the second phase, the test phase, we inoculated sterilized soil with 10% soil inoculum (100 g per pot) conditioned by the different plant species (100% sterilized soil as control) and grew chrysanthemum plants in these soils. With this method the loss of rare microbes should be minimal, as effects are often not observed with dilutions below 100 times (whereas our soil was 10 times diluted) (Kurm *et al*., 2018).

### Expt 1: Microbiomes in inocula vs soil, plant growth and pest colonization

The aim of this experiment was to compare the soil microbiomes of the inocula with those after chrysanthemum had grown in the inoculated soils, as well as the effect of soil inoculation on the growth of plants and resistance to naturally occurring herbivores. As the focus of this study is on soils, and the changes therein by plant legacies, rhizosphere soil was not analyzed, owing to the strong differences with surrounding soil and because it was the surrounding soil that was inoculated and not rhizosphere soil. Field soil was collected and each soil replicate (the different pots during the conditioning phase) was kept separate in this experiment (*n* = 16 soil/plant replicates). Before inoculation, a soil sample was taken from the inocula (first six soil replicates) for microbiome analysis. Plants were grown in a glasshouse under controlled conditions (70% relative humidity, 16 h 21°C : 8 h 16°C, light : dark) and after 3 wk plants were transferred to a semi‐open tunnel glasshouse. This tunnel has a plastic cover and netting on the sides (with a mesh size that allows thrips to enter the glasshouse), and no control of temperature, light or humidity. Here, plants were arranged in a randomized complete block design (one soil replicate per block). The plants of the different soil treatments were randomly distributed inside each block. Plants had no physical barriers between them, and were grown for seven more weeks in the tunnel glasshouse. At the end of the experiment we quantified the density of thrips present on each plant and at each plant height, after which above‐ and below‐ground biomass was clipped and dried at 60°C and DW was determined. During the course of the experiment, we did not observe spatial patterns (i.e. differences in interior and outside plants) in thrips colonization or damage patterns (no data available). Bulk soil was sampled from a subset of six replicate pots per treatment and a subsample from each pot was stored in 2 ml Eppendorf tubes at −80°C to assess the microbiomes in the inoculated soils after chrysanthemum had been grown in the soils.

### Expt 2: Plant and herbivore performance and soil microbiomes

After conditioning the soils, they nwere homogenized per conditioning species and inoculated as described earlier. Ten replicate pots were filled for each of the eight soil inocula and there were 10 pots filled with 100% sterilized soil. After 4 wk of chrysanthemum growth, plant height was measured and three leaves were sampled per plant to evaluate herbivore performance and plant chemistry (see a further description later). At the end of the experiment, the bulk soil of eight pots per soil type was collected and a subsample from each pot was stored in 2 ml Eppendorf tubes at −80°C for later microbiome analysis.

#### Thrips and mite performance

Petri dish plates were prepared with 2 ml of plant agar (1.5%) on one side of the dish. After collecting the leaves, one leaf was placed in each Petri dish, with the petiole inserted into the agar to avoid leaf desiccation, a method shown to be effective to assess resistance to thrips (Maharijaya *et al*., 2015). For each plant, the second fully mature leaf counting from the top of the stem was selected to assess thrips performance, whereas the fourth leaf was selected for spider mite performance. Five thrips larvae (2 d old) were placed on each leaf, and the dishes were sealed with Parafilm and placed in the same climate chambers in which the herbivore was reared. Six days later the number of larvae that reached prepupal stage and total survival were recorded. To evaluate spider mite performance, one female mite was introduced to each Petri dish, and the number of eggs laid by this female was recorded 4 d later.

#### Leaf chlorogenic acid and phenolic acids

The third fully opened leaf was collected from each plant, freeze‐dried and finely ground. Ten milligrams of ground leaf material was then used in a methanol extraction (see Methods S3). In each sample the concentration of chlorogenic acid and of 10 other (unidentified) phenolic compounds was detected using high‐performance liquid chromatography with UV diode array detection (Olszewska, 2007), and quantified based on a chlorogenic acid standard curve (expressed as g^–1^ leaf DW).

### Microbiome analysis

Soil DNA was extracted from the soil samples using the PowerSoil^®^ DNA Isolation Kit according to the manufacturer's instructions (MoBio, Carlsbad, CA, USA). The fungal ITS2 region was amplified using the primers ITS4 and ITS9 (Ihrmark *et al*., 2012) and the bacterial V4 region was targeted using the primers 515F and 806R (Caporaso *et al*., 2012). Amplicons were sequenced on the Illumina MiSeq platform (250 bp paired‐end). Both library preparation and sequencing were done at McGill University and Genome Quebec, Canada.

Fungal sequences were analyzed using the Pipits pipeline (Gweon *et al*., 2015). FunGuild was used to estimate the functions of fungal operational taxonomic unit (OTUs) and the output of this file was compared (curated) against an in‐house database on fungal functions (Nguyen *et al*., 2016; Hannula *et al*., 2017). Bacterial sequences were analyzed using the Hydra pipeline (de Hollander, 2017).

### Statistical analysis

Sequencing data were normalized using the total sum scaling (TSS) (package phyloseq in R) and OTUs occurring in less than three samples with relative abundances of < 0.01% were removed. Furthermore, samples with < 1000 or > 50 000 reads were removed from the dataset. Effects of plant species identity and plant functional group on the structure of the bacterial and fungal community were then examined using PERMANOVA based on a Bray–Curtis dissimilarity matrix in R (package vegan) separately for both experiments. Separations among treatments were visualized using nonmetric multidimensional scaling of a Bray–Curtis dissimilarity matrix. The Chao1 Richness index was calculated for each sample and the effects of inoculation, soil inocula and plant functional group on richness were evaluated using linear models in R (mixed models for inoculation and functional group with soil as random factor as described in the following). The relative abundances were calculated as the number of reads of an OTU, class or phylum divided by the total number of reads in that sample. Pearson correlations were calculated between plant and herbivore parameters, and relative abundances of bacterial phyla and fungal classes using Bonferroni correction for false discovery rate (package corrplot in R).

The design of the experiment with different inocula and one set of control plants (uninoculated plants) prohibits all questions being answered with one statistical model. Therefore, plant and herbivore data were analyzed in three steps (with different models). In the first step, we assessed the effect of inoculation *per se* as the overall effect. For this, data were analyzed with mixed models, with inoculation as a fixed factor and the different soil inocula as random factors. In the second step, we tested differences between the soil treatments. In this analysis, soil inocula (eight inocula plus sterilized soil) were included as fixed factors. Whether each soil inoculum differed from sterilized soil was tested with *post hoc* Dunnett tests using the ‘glht’ function of the multcomp package in R. In the third step, we assessed whether there were differences between inocula originating from grasses and forbs. For this last step we excluded the sterilized soil treatment from the analysis. We used a mixed model with functional group as a fixed factor and soil inocula as a random factor. For Expt 2, in two Petri dishes, six thrips were discovered while only five were introduced (one in a replicate from GM and one in a replicate from LP). These data were excluded from analysis. For the mite bioassay, those plates where no eggs were oviposited were removed because of probable misidentification of females or mortality of females. This resulted in four to 10 replicates per treatment for the mite bioassay.

Data on plant height, biomass and chlorogenic acid were log‐transformed and analyzed with linear models, either with the ‘lm’ or the ‘lme’ (when random factors) function of the nlme package. Data on the proportion of thrips reaching the prepupal stage were analyzed using a generalized linear model with binomial distribution using the functions ‘glm’ or ‘glmer’ (when random factors) of the lme4 package. Data on counts of mite eggs or thrips were overdispersed and therefore analyzed with a generalized linear model with quasi‐Poisson distributions using the functions ‘glm’ or ‘glmmadmb’ (when random factors) from the package glmmadmb. Data from Expt 1 were all analyzed with mixed models where block was set as random factor (in addition to other random factors, as described earlier). All analyses were performed in R v.3.3.3 (R Core Team, 2017).

### Data availability

Paired‐end DNA sequencing reads for this project have been deposited in the European Nucleotide Archive under accession number PRJEB35722 (http://www.ebi.ac.uk/ena/data/view/PRJEB35722). Plant, herbivore and soil chemistry data that support the findings of this study are openly available in Datavers at https://hdl.handle.net/10411/OIQCZH.

## Results

### Microbiome composition from inocula and chrysanthemum soil

**Figure 1:**
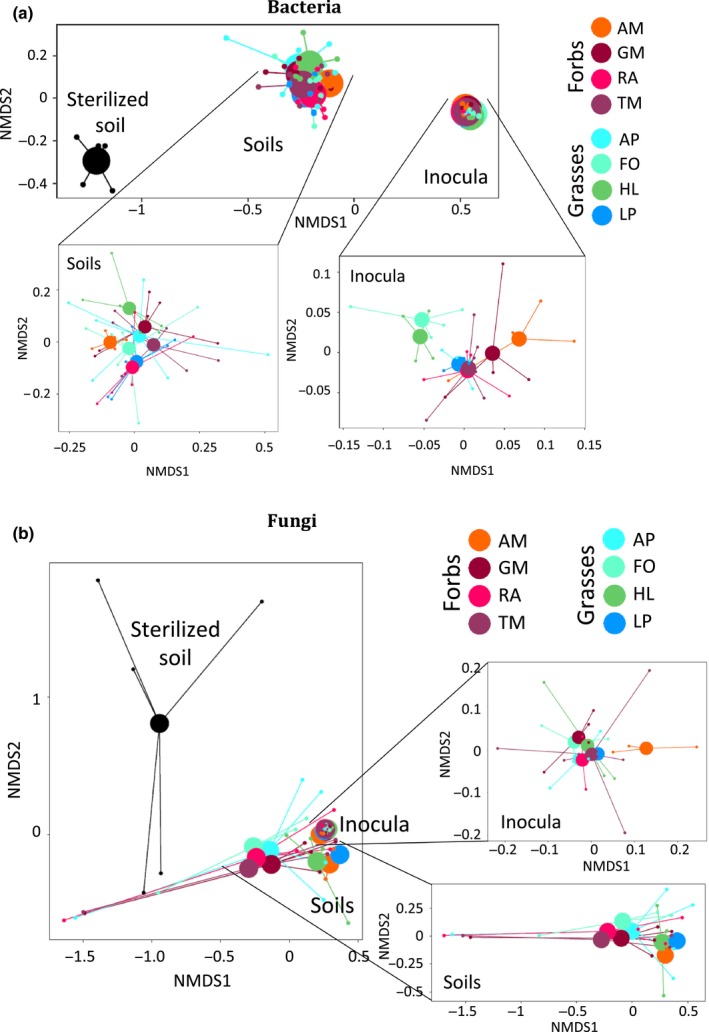
Community structure for bacteria (a) and fungi (b) in the inocula and in the inoculated soils after chrysanthemum growth, colored by plant species (sterilized control in black) in Expt 1. Centroids are shown as large dots and lines connecting the individual samples to the centroids. Inocula were conditioned by grasses (AP, *Alopecurus pratensis*; FO, *Festuca ovina*; HL, *Holcus lanatus*; LP, *Lolium perenne*) or forbs (AM, *Achillea millefolium*; GM, *Galium mollugo*; RA, *Rumex acetosella*; TM, *Tripleurospermum maritimum*).

Our first aim was to assess how the bacterial and fungal communities in the soil change compared with the inocula, once chrysanthemum grew in that soil. For this we sequenced soils from both inocula and chrysanthemum soil from Expt 1. We detected that the community composition for both bacteria and fungi was most affected by whether they were from inocula or chrysanthemum soil, with stronger differences for bacteria than for fungi (PERMANOVA for fungi, *F* = 10.27, *R* ^2^ = 0.15, *P* < 0.001; for bacteria, *F* = 63.79, *R* ^2^ = 0.42, *P* < 0.001; Fig. 1). Furthermore, uninoculated sterilized soils differed greatly from the inoculated soils in terms of microbial communities and were excluded from the multivariate analysis of the soils (Fig 1). For bacteria, the plant species and functional group had a significant effect on the community composition in both the inocula (PERMANOVA; plant species, *F* = 1.32, *R* ^2^ = 0.27, *P* < 0.001; functional group, *F* = 1.77, *R* ^2^ = 0.05, *P* < 0.001) and in the soils after chrysanthemum had grown in them (plant species, *F* = 1.65, *R* ^2^ = 0.22, *P* < 0.001; functional group, *F* = 1.69, *R* ^2^ = 0.04, *P* = 0.023; Fig. 1). By contrast, fungal communities were not affected by plant species or functional group in the inoculum (PERMANOVA; plant species, *F* = 0.99, *R* ^2^ = 0.29, *P* = 0.52; functional group, *F* = 1.12, *R* ^2^ = 0.05, *P* = 0.27) or in the soils (plant species, *F* = 0.66, *R* ^2^ = 0.19, *P* = 0.96; functional group, *F* = 1.07, *R* ^2^ = 0.04, *P* = 0.37). Analyses of microbial richness in inocula and chrysanthemum soils, as well as a description of community composition and richness from the second experiment are shown in Notes S1 and Figs S1, S2.

### Chrysanthemum effect on bacterial and fungal OTUs

**Figure 2:**
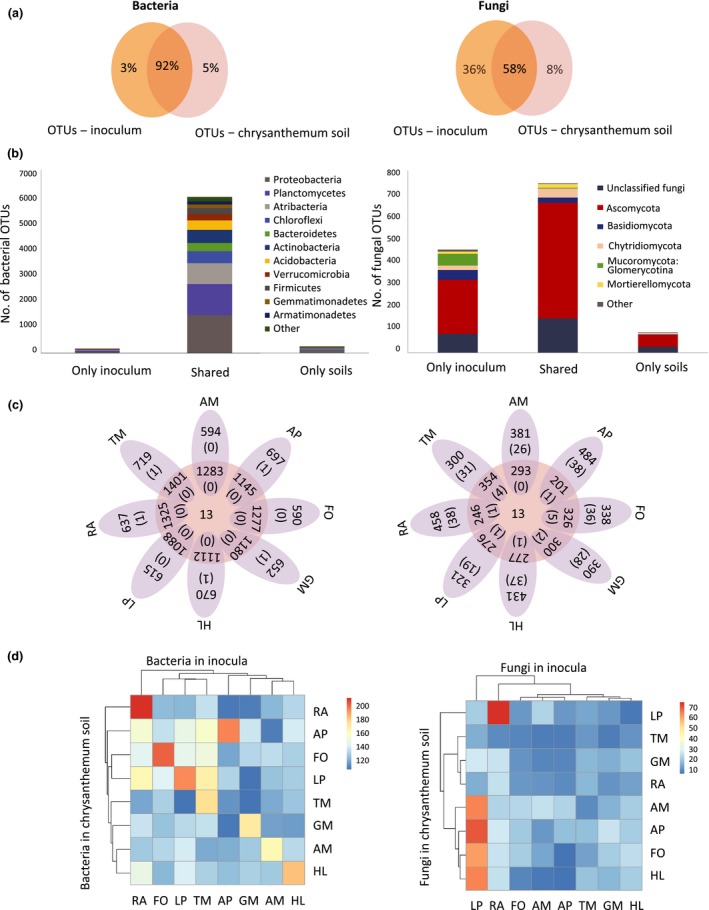
Bacterial (left side) and fungal (right side) operational taxonomic units (OTUs) shared among inocula and inoculated soils after chrysanthemum growth. (a) Venn diagrams of OTUs found (%) in the overall soil inocula or after chrysanthemum growth. (b) Total number of unique and shared OTUs of inocula and soil after chrysanthemum growth, depicted at phylum/class level. (c) Venn diagrams showing total numbers of OTUs found in the different soil inocula (oval sides) or after chrysanthemum growth (round center). The number of unique OTUs is shown in parentheses. (d) Shared OTUs between plant species in inocula and soils, clustered by similarity. Inocula were conditioned by grasses (AP, *Alopecurus pratensis*; FO, *Festuca ovina*; HL, *Holcus lanatus*; LP, *Lolium perenne*) or forbs (AM, *Achillea millefolium*; GM, *Galium mollugo*; RA, *Rumex acetosella*; TM, *Tripleurospermum maritimum*).

We further investigated the shared proportion of OTUs between inocula and chrysanthemum soils from Expt 1. For bacteria there was a reduction from 5000 OTUs in the inocula to *c*. 4200 OTUs in the soil, and for fungi a reduction from 220 to 110 phylotypes. For bacteria, 92% of the total OTUs were found both in the inocula and in the soil after growth of chrysanthemum (Fig. 2a), whereas only 3% of the OTUs were present in the inoculum and these were not found later in the soil. For fungi the situation was quite different, as only 58% of total phylotypes were shared between the inocula and soils and 36% of the phylotypes were lost (only present in the inocula). After chrysanthemum growth, 5% of total bacterial OTUs and 8% of fungal phylotypes were detected that were not present in the inocula. Remarkably, out of 55 phylotypes belonging to the phylum Mucoromycota (including the subphylum Glomeromycotina with arbuscular mycorrhizal fungi) present in the inocula, only four were detected in the soils after chrysanthemum growth (Figs 2b, S3). For bacteria, almost none of the OTUs were unique for a single inoculum (Fig. 2c), while for fungi, an average of *c.* 8% of the phylotypes detected in each inoculum type were unique. However, this uniqueness in fungi in the inocula is almost lost after chrysanthemum growth (Fig. 2c). Visualizing the similarity between all inocula and the soils after chrysanthemum growth reveals that for fungi not many phylotypes are shared between inocula and soils (Fig. 2d), with the exception of LP inoculum, which contained a high number fungal phylotypes that were shared especially with soils inoculated with AM, AP, HL and FO. For bacteria, all inocula shared a high similarity in terms of shared OTUs with the soil where the same inoculum was introduced. RA and FO soils shared the most OTUs with their respective inocula.

### Soil inoculation effects on plant growth, resistance to herbivores and plant defenses

**Figure 3:**
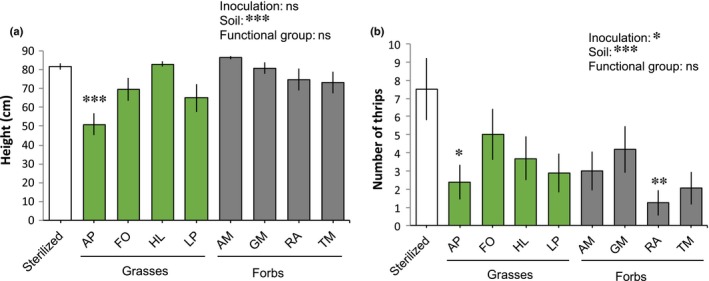
(a, b) Effects of soil inoculation on chrysanthemum height (a) and thrips numbers (b) in Expt 1 with full plants in a semi‐open glasshouse with natural thrips colonization. Sterilized soil was inoculated with 10% sterile soil or soil conditioned by grasses (AP, *Alopecurus pratensis*; FO, *Festuca ovina*; HL, *Holcus lanatus*; LP, *Lolium perenne*) or forbs (AM, *Achillea millefolium*; GM, *Galium mollugo*; RA, *Rumex acetosella*; TM, *Tripleurospermum maritimum*). Bars represent means ± SE (*n* = 16 plants). These parameters are analyzed for the effect of overall inoculation (inoculated or sterilized), specific soil inocula (eight conditioned soils plus sterilized), and functional group (grasses or forbs, excluding sterilized); asterisks above bars indicate significant differences with the sterilized soil (Dunnett test). ***, *P* < 0.001; **, *P* < 0.01; *, *P* < 0.05; ns, not significant.

In Expt 1, conducted in a semi‐open tunnel glasshouse where thrips were naturally present, chrysanthemum height was not affected by soil inoculation overall (Fig. 3a). However, there were differences between specific soil inocula, with plants growing in soil conditioned by the grass AP being smaller than plants grown in sterilized soil. Total chrysanthemum biomass showed a similar pattern to plant height, but in this case also plants growing in soil conditioned by the grass LP were smaller than plants growing in 100% sterilized soil (Table 1; Fig. S4). There were no significant plant functional group effects on plant height. The number of thrips on chrysanthemum plants was strongly reduced by soil inoculation (Fig. 3b). Fewer thrips were observed on plants growing in soil conditioned by the grass AP and the forb RA than on plants growing in sterilized soil. The functional group of the plants that conditioned the inocula, however, did not affect the number of thrips on chrysanthemum.

**Table 1:** Results of the statistical analyses of the plant and herbivore parameters.

	Inoculation			Soil inocula			Functional group		
	*F*/χ^2^ a	df1; df2^b^	*P*	*F*/χ^2^	df1; df2	*P*	*F*/χ^2^	df1; df2^b^	*P*
Expt 1: outdoor									
Thrips	5.29	1	0.02	70.58	8	< 0.001	1.29	1	0.257
Total biomass	1.25	1; 7	0.301	5.53	8; 120	< 0.001	1.46	1; 6	0.273
Height	0.69	1; 7	0.432	4.19	8; 120	< 0.001	2.32	1; 6	0.178
Expt 2: detached leaf assays									
Height	0.223	1; 7	0.651	3.05	8; 81	0.005	0.66	1; 6	0.449
Thrips pupation	4.68	1	0.030	20.12	8; 79	0.009	2.65	1	0.104
Thrips survival	3.06	1	0.080	15.21	8; 79	0.055	2.61	1	0.106
Spider mite eggs	1.23	1	0.268	40.6	8; 52	0.816	0.08	1	0.783
Chlorogenic acid	2.95	1; 7	0.129	3.10	8; 80	0.004	0.50	1; 6	0.505
Phenolics	1.21	1; 7	0.309	3.42	8; 80	0.002	1.79	1; 6	0.229

a *F*‐values are given for linear models; whereas χ^2^ are given for generalized linear models.

b When a generalized linear mixed model was used, no residual df is given because of computational issues (Skaug *et al*., 2013).

Fungal richness was not related to plant height (Table 2) in Expt 1. By contrast, bacterial richness in chrysanthemum soil explained 17% of the variation of plant height, whereas bacterial richness in the inocula did not significantly explain plant height. Additionally, this positive correlation was driven by those soils containing forb inocula where bacterial richness explained 37% of the variation in plant height, whereas in grass‐inoculated soil, bacterial richness was not correlated with plant performance. The relationship between the number of thrips and the soil microbiome could not be analyzed for Expt 1 as a result of very low numbers of thrips on the subset of plants that were included for microbiome analysis.

**Table 2:** Results of Pearson correlations between chrysanthemum height from Expt 1 and bacterial and fungal richness (Chao1) in inocula conditioned by grasses or forbs, and their respective inoculated soil after chrysanthemum growth.

	Bacteria		Fungi	
	*R* ^2^	*P*	*R* ^2^	*P*
Grass inocula	0.11	0.23	0.11	0.36
Forb inocula	0.06	0.35	0.01	0.77
Total inocula	0.01	0.65	0.02	0.57
Grass soil	0.10	0.26	0.16	0.24
Forb soil	0.37	0.008**	0.11	0.16
Total soil	0.17	0.02*	0.04	0.37

Asterisks highlight significant correlations: **, *P* < 0.01; *, *P* < 0.05.

**Figure 4:**
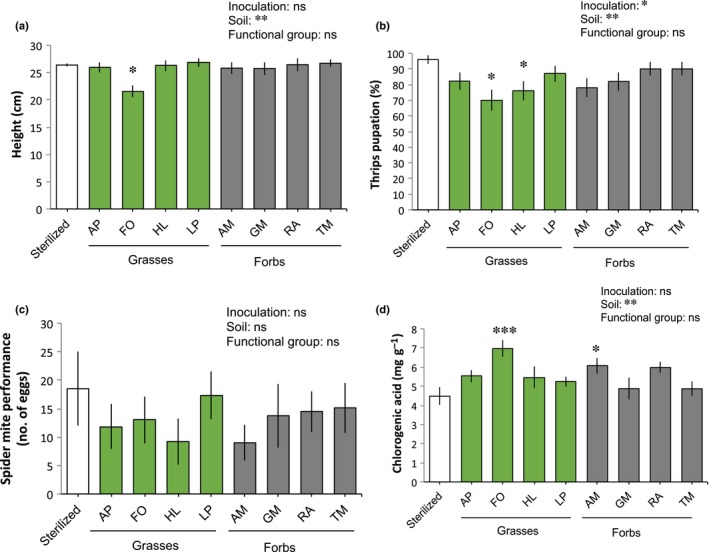
(a–d) Effects of soil inoculation on chrysanthemum height (a); thrips (b) and spider mite (c) performance; and chlorogenic acid concentrations in plants (d) in Expt 2. Sterilized soil was inoculated with 10% sterile soil or soil conditioned by grasses (AP, *Alopecurus pratensis*; FO, *Festuca ovina*; HL, *Holcus lanatus*; LP, *Lolium perenne*) or forbs (AM, *Achillea millefolium*; GM, *Galium mollugo*; RA, *Rumex acetosella*; TM, *Tripleurospermum maritimum*). Plants were grown in a glasshouse and herbivore performance was assessed in detached leaf assays. Bars represent means ± SE (*n* = 10 plants; panel (c): four to 10 replicates; estimated means for generalized linear models). These parameters are analyzed for the effect of overall inoculation (inoculated or sterilized), specific soil inocula (eight conditioned soils plus sterilized), and functional group (grasses or forbs, excluding sterilized); asterisks above bars indicate significant differences with the sterilized soil (Dunnett test). ***, *P* < 0.001; **, *P* < 0.01; *, *P* < 0.05; ns, not significant.

**Figure 5:**
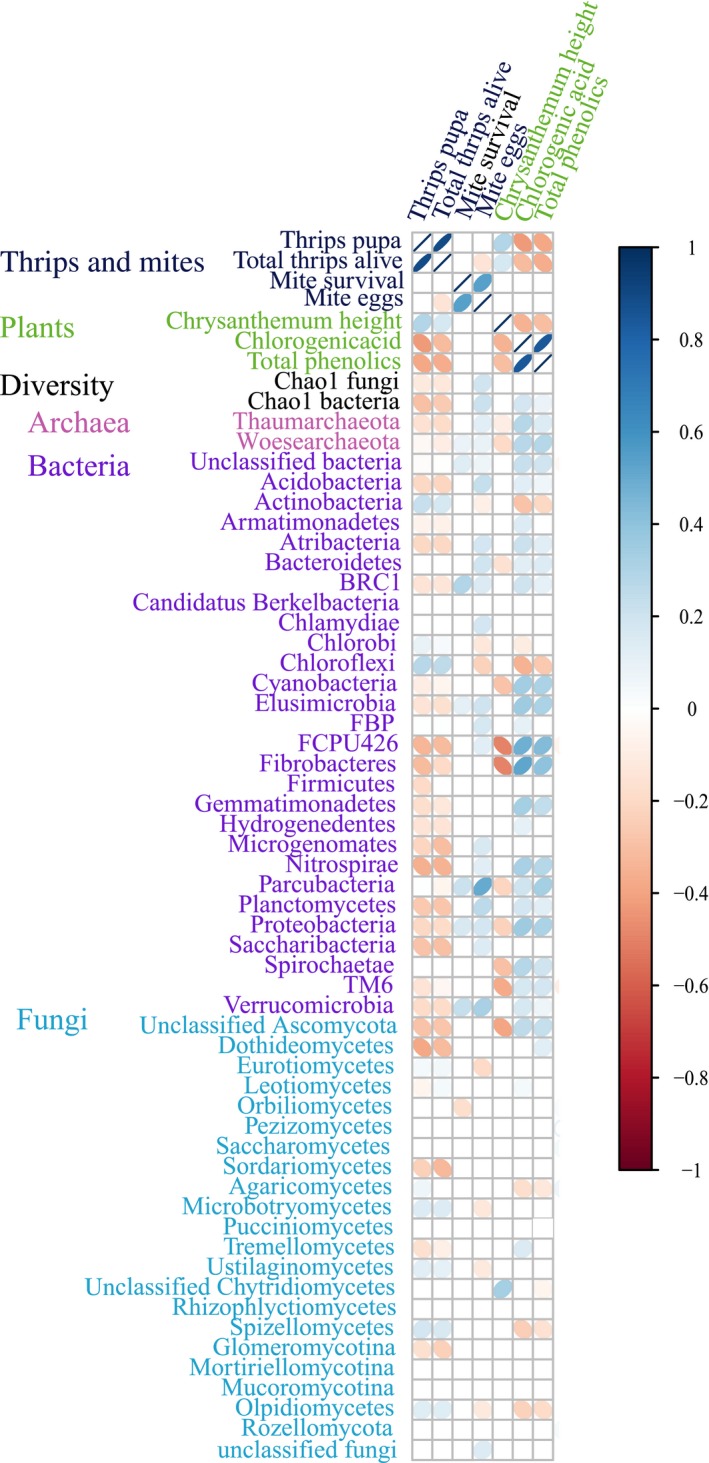
Correlations between parameters of plant performance and resistance and relative abundance of fungal and bacterial taxa in the soil after chrysanthemum growth from Expt 2. The scale color of the filled squares indicates the strength of the linear Pearson correlation coefficients (*r*) and whether it is negative (red) or positive (blue). Only significant correlations with *P* < 0.05 after Bonferroni correction are shown. If the correlation is not significant, the box is left white.

In Expt 2, in a more controlled environment and with detached‐leaf assays, the percentage of thrips reaching the pupal stage was reduced both by soil inoculation overall and by specific soil inocula, but was not affected by the functional group (grasses or forbs) of the conditioning plants. The percentage of thrips reaching the pupal stage was significantly lower on plants growing in soil conditioned by the grasses HL and FO compared with sterilized soil (Fig. 4b). Final thrips survival followed a similar trend as the number of thrips reaching the pupal stage, although in this case the effects of soil inoculation and specific soil inocula were not statistically significant (*P* = 0.08 and *P* = 0.055, respectively; Table 1; Fig. S5). In contrast to the effects on thrips, soil inoculation, specific soil inocula and functional group did not affect the performance of spider mites, measured as the number of eggs laid in a period of 4 d, even though egg production was generally lower on plants growing in inoculated than in sterilized soil (Fig. 4c). In Expt 2, the overall effect of soil inoculation, including the eight species‐specific inocula, or the functional group of the conditioning plants did not affect chrysanthemum height or foliar concentrations of chlorogenic acid (Fig. 4). However, when testing the effects of species‐specific inocula, chlorogenic acid concentrations were higher in plants that grew in inoculated soil conditioned by FO (and plant height was the lowest) and AM than in plants growing in sterilized soil (further results on effects of species‐specific inocula are described in Notes S2; Fig. S6). We also found that the concentrations of chlorogenic acid (and total phenolics) were negatively correlated with thrips performance (thrips reaching the pupal stage, *R* = −0.42, *P* = 0.001; final thrips survival, *R* = −0.32, *P* = 0.018; Fig. 5).

### Correlations of microbial groups with plant performance and resistance to herbivores

Last, we explored the relationship between relative abundances of bacterial and fungal taxa in the soil and plant performance and resistance parameters from the second experiment. Plant height was negatively correlated with eight bacterial phyla (strongest for FCPU426 and Fibrobacteres, and one taxa therein; Fig. S7) and with unclassified Ascomycota (Fig. 5). Only one fungal class was positively correlated with plant height (unknown Chytridiomycetes). Chlorogenic acid and total content of phenolic acids were treated together, as the two parameters were strongly correlated (*r* = 0.85, *P* < 0.001). Most bacterial phyla (20 out of 28 bacterial phyla) were positively correlated with the chlorogenic acid content of the plant while only three bacterial phyla were negatively correlated with the chlorogenic acid content (Fig. 5). The relative abundance of three fungal classes was negatively correlated with chlorogenic acid and the relative abundance of two fungal classes was positively correlated with chlorogenic acid. These correlations were, however, all weaker than those for detected for bacteria.

The relative abundance of 17 bacterial phyla was significantly negatively correlated with thrips survival and the number of pupae, while two phyla were positively correlated with thrips survival (Fig. 5). The bacterial groups with strongest correlation were Chloroflexi, Fibrobacteres, FCPU426, Nitrospirae, Planctomycetes and Saccharibacteria (Fig. S7a). From 22 fungal classes analyzed, five classes were negatively correlated and four were positively correlated with number of surviving thrips. Most strongly, the relative abundance of unclassified Ascomycota and especially members of the class Dothideomycetes were associated with a decrease in numbers of thrips (Fig. S7b). Thrips pupation was positively correlated with plant height but negatively correlated with chlorogenic acid and total phenolics. At the microbial level, thrips pupation was negatively correlated with bacterial richness and, to a lesser extent, with fungal richness. Chlorogenic acid, by contrast, was positively correlated with bacterial richness but not with fungal richness. Most bacterial phyla (and only two fungal classes) correlated negatively with thrips and positively with chlorogenic acid concentrations. The number of mite eggs on the leaves was significantly positively correlated with the relative abundance of 17 bacterial phyla and one fungal class, while the relative abundance of three bacterial phyla and four fungal classes were negatively correlated with the number of eggs (Fig. 5). The strongest positive correlations were detected for Parcubacteria and Verrucomicrobia. The survival of mites was positively related to six bacterial phyla (with the strongest correlation with BRC1) and negatively to one fungal class. Relative abundances of the taxonomic groups reported in Fig. 5 in each sample are shown in Table S1 and correlations between microbial groups are shown in Fig. S8. A more thorough analysis of the microbial groups aiming at genus level showing the strongest correlations is shown in Fig. S9, and the average relative abundances at this level are shown in Table S2. A full description of the richness and community composition of the soil microbiomes from Expt 2 is also presented in Notes S3, Figs S10–S12 and Table S3.

## Discussion

The overall goal of this study was to assess whether applying the concept of plant–soil feedbacks could change the soil microbiome to a state that induces resistance to above‐ground herbivorous pests in a horticultural crop. Here, we show that soil inoculation with species‐specific conditioned soil leads to strong differences in the microbiome in the soil in which a focal plant grows, both in the overall community composition and in the abundance of specific microbial groups. The functional group of the plant species that conditioned the soil used as inoculum did not affect chrysanthemum growth, resistance to herbivores, or different aspects of the fungal communities in our study. However, it influenced bacterial community assembly both in soils after chrysanthemum growth, and in the original inocula, and it influenced the relationship between bacterial richness and chrysanthemum growth, and also how chrysanthemum affected soil bacterial richness. We show that inoculation with soils in which previously wild grasses and forbs were growing can induce resistance in above‐ground plant tissues to thrips, but not mites, both in detached leaf assays and in semi‐field conditions. We further found a correlation of thrips resistance and of chlorogenic acid with bacterial and fungal richness. However, although the effect of overall inoculation consistently induced resistance, our study also shows that the effect that a specific plant species has on chrysanthemum growth and thrips performance via changes in the soil inoculum is variable. This is not surprising when considering the diversity of strains, and therefore functions present in the soil, and the interactions between them.

We hypothesized that every plant species would exert a specific effect on the microbiome in their soil. An important question is how this is maintained when another plant species grows in that soil later, as the latter plant also influences the soil microbiome (Ma *et al*., 2018; Wubs & Bezemer, 2018). Here we show that chrysanthemum exerted a strong negative effect on fungi (especially on the Glomeromycotina, known as arbuscular mycorrhiza), as only 58% of the total fungal OTUs were present both in the inocula and in the soil after chrysanthemum growth (Fig. 2b). Although there were clear differences among the bacterial communities of the different inocula, among the inocula and the inoculated soils after chrysanthemum growth, between grasses and forb inocula, these were more resilient to the effect of chrysanthemum growth, with > 90% of bacterial OTUs shared between inocula and inoculated soil in which chrysanthemum had grown (Fig. 2). The effect of chrysanthemum on mycorrhizal fungi was unexpected, as previous studies have shown colonization by mycorrhiza of other cultivars of chrysanthemum (Wang *et al*., 2018). However, later work by our group supported our findings after observing < 1% mycorrhizal root colonization with staining techniques (H‐K. Ma *et al*., unpublished). Recent studies have shown that the genetic background of the test plant can determine the root‐associated microbiome and whether conspecific plant–soil feedback effects on plant growth are negative or neutral (Hu *et al*., 2018; Carrillo *et al*., 2019). This has been an oft‐overlooked aspect during the breeding process (Pérez‐Jaramillo *et al*., 2016; Carrillo *et al*., 2019), and here we show that a certain crop or cultivar may inhibit beneficial microbial groups such as mycorrhiza.

Grasses and forbs shaped distinct bacterial communities in the soil and these differences remained after chrysanthemum had been grown in soils inoculated with those communities (Fig. 1). Remarkably, there was an unexpected strong negative correlation between bacterial richness in the inoculum and in the inoculated soil after chrysanthemum growth for grass‐conditioned inocula but not for forb‐conditioned ones (Fig. S2). We hypothesize that by shaping bacterial communities in the soil, grasses may enrich the soil with grass‐associated microbes and simultaneously reduce the richness (e.g. by competition or antibiosis) of forb‐associated microbes. This means that when a forb (chrysanthemum in this case) grows later in that soil, a higher grass‐associated richness in the inoculum leads to a lower forb‐associated richness in the soil after the forb has grown in this soil. Supporting this hypothesis, chrysanthemum height was strongly positively correlated with the diversity of bacteria (but not fungi) in chrysanthemum soil; however, this was only true for forb‐conditioned inocula, and not for grass‐conditioned inocula (Table 2). Soil microbial diversity is a main driver of plant productivity under certain scenarios (Wagg *et al*., 2014; Delgado‐Baquerizo *et al*., 2016), and we show here that this will depend on the functional group of the plant that previously grew in that soil. Whether these differences between grass and forbminocula are a general pattern that differs with the functional group of the focal plant is something that needs more attention.

Based on studies of plant interactions with individual microbial strains, plant resistance to above‐ground herbivores is a trait that is partially mediated by the plant's symbiosis with specific soilborne microbes, known as microbial‐induced systemic resistance (ISR) (Pineda *et al*., 2010; Jung *et al*., 2012; Pangesti *et al*., 2017). Here we show that plant resistance to herbivores also depends on the whole soil microbiome, which can be inoculated to enhance such resistance (Figs 3, 4). However, we cannot exclude the possibility that, inside this complex soil microbiome, the observed ISR could have been triggered by a single microbial strain. In a microbiome context, the little evidence for whole microbiome ISR that is available so far in the literature is mostly for leaf chewers such as caterpillars or leaf beetles (Badri *et al*., 2013; Hubbard *et al*., 2019). Until now, it was unknown if microbiome ISR could also be effective against cell‐feeding herbivores such as thrips. Despite their importance as worldwide pests, little information is available about the potential role of soilborne microbes in reducing thrips populations. Several studies have shown, however, that fungal endophytes (that inhabit the soil) reduce the performance of *Thrips tabaci* and can reduce virus incidence transmitted by thrips (Muvea *et al*., 2014; Muvea *et al*., 2018). In our study, we identified 11 bacterial phyla – especially Nitrospirae and Planctomycetes – and one fungal class that were excellent candidates of beneficial microbes, that is, their relative abundances were negatively correlated with thrips and positively correlated with chlorogenic acid without impacting plant growth (Fig. 5). Although these results are correlative, this opens a new field to explore the role of those specific taxa inside the soil microbiome and their effects on plant defenses.

When analyzing the overall effect of inoculation, no effect was observed on plant performance, suggesting that the effects of inoculation on herbivores are not a result of a direct relationship between plant growth and herbivore fitness and that they may be related to plant defenses. Especially interesting are those inocula that led to a reduction in thrips without affecting plant growth, which was the case for soil inocula that consisted of RA‐ or HL‐conditioned soil (Figs 3, 4). We expected that, in addition to increasing resistance, the selected species and especially grasses would promote chrysanthemum growth, based on our previous studies where chrysanthemum grew better in soils with those inocula than in sterilized soils (Ma *et al*., 2017, 2018). A possible explanation for the lack of plant growth promotion in inoculated soil in this study is that even for a single microbial strain, a common duality that is observed is that the establishment of symbiosis has a cost for the plant that might result in reduced growth (Morgan *et al*., 2005), contributing to a spectrum of positive, neutral and negative effects of microbes. At the negative side of this spectrum, pathogen accumulation seems a common mechanism for negative plant‐soil feedbacks (PSFs), especially between plants that belong to the same species (Hu *et al*., 2018; Wang *et al*., 2019). Here we observed a reduction in plant growth and an increase in concentrations of chlorogenic acid in the leaves of plants growing in soil inoculated with FO (Expt 2; Fig. 4). Additionally, we found microbial groups, especially Fibrobacteres, FCPU426 and unclassified Ascomycota, that, besides being negatively correlated with thrips and positively with chlorogenic acid, were also negatively correlated with plant growth (Fig. 5). Chlorogenic acid has a defensive function and it can be induced in response to below‐ and above‐ground pathogens (Atanasova‐Penichon *et al*., 2012; Ma *et al*., 2017), but we cannot discern here whether the lack of plant growth promotion is a result of the presence of pathogens or to the general costs of the symbiosis. In a microbiome context where the plant must establish a dialogue with a multitude of strains, it is not surprising that this cost increases and that the benefits of the soil microbiome may only pay off under stressful scenarios.

This idea is in line with the priming concept where the plant defensive response is primed by microbes but then only mounted after attack or stress. Accordingly, the benefit of the microbial symbiosis on plant fitness is only evident in the presence of the attacker (van Hulten *et al*., 2006; Martínez‐Medina *et al*., 2016). Our study was not designed to assess the priming mechanism, and plant height and chlorogenic acid were measured before herbivore attack. Future work with model systems for which molecular tools are available could confirm the role of defensive compounds in microbiome ISR and priming. Therefore, the changes in chlorogenic acid might be even stronger in a real scenario where plants are responding to their attackers, as has been previously shown for plants colonized with mycorrhizas and attacked by pathogens (Sanchez‐Bel *et al*., 2016). Also important is the fact that microbiomes are an intrinsic component of the plant as a holobiont, providing plants with an extended phenotype (Vandenkoornhuyse *et al*., 2015). Inside a microbial community, where some strains might contribute to nutrient acquisition, others may enhance tolerance to drought, and still others resistance to pathogens. Here we isolated the effects on herbivorous insects by performing the experiments in controlled conditions with abundant water, nutrients and a relative absence of pathogens. Based on this, we expect that the effects reported could be amplified in field conditions in the presence of other biotic and abiotic stresses, as it has also been suggested for the general effects of PSFs (De Long *et al*., 2019).

In conclusion, we show that soil inoculation and the application of plant–soil feedbacks to create different soil microbiomes comprise a strategy that can reduce pest incidence in above‐ground tissues. Although the application of plant–soil feedbacks for pest control via influencing the soil microbiome had been suggested previously, empirical evidence from agricultural systems has been lacking so far. A major challenge is how to select conditioning plants that create beneficial soil microbiomes that consistently reduce pests and promote plant growth, within the context of highly diverse and variable soil microbiomes. Hence, the ‘holy grail’ in research on microbiome‐induced plant resistance is to find plant species that modify the soil microbiome in a predictable and desirable way. Following a top‐down approach, the concept of plant–soil feedbacks could also be used as a source of discovery of keystone microbial taxa that induce resistance in plants. This study contributes to the necessary change in the paradigm of agricultural practices, where in addition to focusing on reducing negative plant–soil feedbacks and pathogenic microbes, more attention should be paid to making use of positive PSFs and beneficial microbes. Ecologically based strategies are needed to improve the sustainability of our agricultural systems, and our study emphasizes that the soil is a key component.

## Author contributions

AP, IK and TMB design the research; AP, IK and WG performed the research and collected the data; AP, IK, SEH and TMB analyzed and interpreted the data; AP and SEH wrote the initial version of the manuscript and all authors contributed to its revision.

## Supporting information

Please note: Wiley Blackwell are not responsible for the content or functionality of any Supporting Information supplied by the authors. Any queries (other than missing material) should be directed to the *New Phytologist* Central Office.

**Fig. S1** Richness of bacteria and fungal phylotypes in the inocula (upper panels) and in the soil after chrysanthemum growth (bottom panels).
**Fig. S2** Correlations between the richness (Chao1 index) of bacteria (left) and fungi (right) in the inocula and in the soils after chrysanthemum growth in Expt 1.
**Fig. S3** Richness (Chao1) of fungal phylotypes of the subphylum Glomeromycotina.
**Fig. S4** Effects of soil inoculation on chrysanthemum biomass of plants from Expt 1.
**Fig. S5** Effects of soil inoculation on thrips survival from Expt 2.
**Fig. S6** Effects of soil inoculation on Phenolics in leaves from uninfested plants from Expt 2.
**Fig. S7** The relative abundance of bacterial (a) and fungal (b) groups showing the strongest correlation with thrips performance in Expt 2.
**Fig. S8** Correlations between parameters of plant performance and resistance and relative abundance of fungal and bacterial taxa in Expt 2.
**Fig. S9** Correlations at the genus level between parameters of plant performance and resistance and relative abundance of fungal and bacterial taxa in Expt 2.
**Fig. S10** Bacterial and fungal richness (top panels) and community composition (bottom panels) in the soil after chrysanthemum growth from Expt 2.
**Fig. S11** The relative abundance of bacterial phyla that were affected by the soil conditioning and inoculation in Expt 2.
**Fig. S12** The relative abundance of fungal phyla that were affected by the soil conditioning and inoculation in Expt 2.
**Methods S1** Herbivore rearing.
**Methods S2** Soil preparation and plant growth.
**Methods S3** Chemical analysis of phenolics.
**Notes S1** Soil inoculation effects on microbial richness in inocula and soils.
**Notes S2** Soil inoculation effects on plant growth and plant defenses.
**Notes S3** Microbial richness and community composition of the chrysanthemum soils from Expt 2.

**Table S1** Data used to make Figs 5 and S9 which show correlations between parameters of plant performance and resistance and relative abundance of fungal and bacterial taxa.

**Table S2** Average relative abundances of bacteria and fungi at the lowest taxonomic level that could be identified in chrysanthemum soil and inocula.

**Table S3** Effect of different factors on the relative abundances of bacterial and fungal taxonomic groups.
