# Supplementary material for: Conditioning the soil microbiome through plant–soil feedbacks suppresses an aboveground insect pest
Source: New Phytol. 2020 Feb 3;226(2):595–608. doi: 10.1111/nph.16385 (PMC7155073; doi:10.1111/nph.16385)

New Phytologist

New Phytologist **Supporting Information**

Article title: **Conditioning the soil microbiome through plant-soil feedbacks suppresses an aboveground insect pest**
Authors: Ana Pineda, Ian Kaplan, S. Emilia Hannula, Wadih Ghanem & T. Martijn Bezemer
Article acceptance date: 04 December 2019

The following Supporting Information is available for this article:

Methods S1-S3

Methods S1 Herbivores rearing

Thrips were reared for multiple generations on pods of Romano beans (*Vicia faba*) purchased weekly in a local supermarket. Thrips were reared in 0.7 l glass jars with anti-thrips mesh glued to the screw-cap top. To obtain first-instar larvae to use in the experiments, batches of eggs that were laid during a 24 h-period were collected. Mites were reared on detached leaves of Lima bean plants (*Phaseolus vulgaris* cv. Speedy), on trays containing wet cotton. To obtain a cohort of similar aged females, 18 days before the experiment, adults were individually transferred to uninfested leaves for two days and then removed. With this method we obtained the so-called egg waves of a similar age (± 1 day), of which we later selected females to be used in the experiments . Thrips and mites were reared in separate climate chambers with a 16 h light and 8 h dark photo regime with 25°C for mites and 22°C for thrips.

Methods S2 Soil preparation and plant growth

a) Phase 1: Soil conditioning Field soil was collected (5–20 cm below the surface) from a natural grassland (Mossel, Ede, Netherlands), sieved (1 cm mesh size) to remove coarse fragments and macro-arthropods, and homogenized. To obtain the different soil inocula, two one-week-old seedlings of a single plant species were transplanted into each pot (11 x 11 x 12 cm, with 1 kg of

soil). Seeds were obtained from a wild plant seed supplier (Cruydt-Hoeck, Assen, The Netherlands). Seeds were surface sterilized—1 min in 1% sodium hypochlorite solution and rinsed with distilled water afterwards—and germinated on sterile glass beads in a climate chamber at 20°C/15°C (16 h/8 h, light/dark). Seedlings that died within 7 days were replaced once. Pots were randomly placed in a greenhouse with controlled conditions: 70% RH and 16 h light (21°C) /8 h dark (16°C) photo regime. Natural daylight was supplemented with 400 W metal halide lamps (225 µmol s-1 m-2 photosynthetically active radiation, 1 lamp per 1.5 m2). Plants were checked three times per week and watered as needed. Two months after transplanting, soil was collected by hand-shaking the full root system. Finer roots were kept in the soil because densities of microorganisms are highest in the rhizosphere. Soil from each pot (10-20 pots per species, depending on the experiment) was homogenized and stored individually in closed plastic bags at 4°C until used in the test (feedback) phase. These soils are called "soil inocula" hereafter. b) Phase 2: Inoculation and plant growthSeveral hundred kg of soil were collected from the same area as the soil for conditioning. After sieving and homogenizing, the soil was sterilized by gamma irradiation (>25 K Gray gamma irradiation, Isotron, Ede, Netherlands). Pots (11 cm × 11 cm × 12 cm) were filled with one kg of a homogenized mixture of 10% soil inoculum as described above and 90% sterilized soil (based on weight). As a control we included pots filled with 100% sterilized soil. Prior to planting, the soil in each pot was well-watered and 100 ml half-strength Hoagland nutrient solution was added. Two chrysanthemum cuttings (without roots) were planted in each pot. The pots were placed on trolleys that were tightly covered with a thin transparent plastic foil for 10 days to create an environment with high humidity that favors rooting. After 10 days, one of the chrysanthemum

cuttings was removed from each pot. Plants were fertilized following grower practices: half-strength Hoagland nutrient solution for the first 2 weeks, and single strength Hoagland solution during the following weeks.

Methods S3 Chemical analysis of phenolics

Each leaf sample was extracted twice. In the first extraction, 1 ml 70% MeOH was added to each sample, vortexed for 0.5 min, then ultrasonicated for 30 min at 20◦C, centrifuged for 10 min at 10000 rpm, and labeled. The extraction was repeated so that each sample was extracted by 2 ml 70% MeOH. The extraction was filtered using a 0.2 µm PTFE syringe filter and stored at −20◦C until analysis. Chemical analysis was performed using high performance liquid chromatography (HPLC) with UV diode array detection. A standard solution that contained 10 mg chlorogenic acid per 10 ml 70% MeOH was diluted to produce an external standard curve.

Notes S1-S3: Results

Note S1 Soil inoculation effects on microbial richness in inocula and soils

In the first experiment we analyzed richness (Chao1 index) as a diversity indicator, and as expected, sterilized soil had a substantially lower bacterial and fungal richness than inoculated ones (bacteria: $F_{1,7}$=127.4, p<0.001, fungi: $F_{1, 7}$=12.4, p=0.009; Fig. S1). When comparing the inoculated soils, there was no effect of conditioning plant species or plant functional group on the richness of fungi or bacteria in the inoculum (Bacteria: plant species $F_{7,25}$= 1.45, p=0.23; functional group $F_{1,6}$= 0.78, p=0.41; Fungi: plant species $F_{7,14}$=0.95, p=0.5; functional group $F_{1,6}$= 0.18, p=0.69; Fig. S1). There was, however, an effect of conditioning plant species identity on

the richness of bacteria ($F_{7,25}$=2.56, p=0.039, Fig. S1) in the soil after chrysanthemum had been grown in it. Especially, inoculating with AM soil leads to higher richness in the soil after growth of chrysanthemum than conditioning with LP (Tukey, $p<0.05$; Fig. S1). For fungi there was no effect of plant species on fungal richness in the soils after chrysanthemum growth ($F_{7,20}$=1.95, p=0.11). Functional group did not affect bacterial ($F_{1,6}$=0.40, p=0.55) or fungal richness ($F_{1,6}$=1.07, p=0.34) in the soil after chrysanthemum had been grown in it.

The microbial richness in the inocula and in the soil after chrysanthemum had been grown in them were not correlated (Fig. S2). For bacteria this was partly explained by differential effect of forbs and grasses: there was a negative correlation between the richness of bacteria in the inocula and the richness of bacteria in the soils for grasses (R^2= 0.37, p=0.015) but not for forbs (Fig. S2). For fungi, for both functional groups there was no correlation between richness in the inocula and richness in the soil after chrysanthemum growth.

Note S2 Soil inoculation effects on plant growth and plant defenses

In experiment two, prior to leaf sampling for the herbivore assay, chrysanthemum height was not affected by soil inoculation, nor by the functional group of the conditioning plants (Fig. 4a). However, specific soil inocula did affect height, with plants growing in soil conditioned by the grass *Festuca ovina* (FO) being smaller than plants grown in 100% sterilized soil. Inoculation with the other seven conditioned soils did not affect chrysanthemum height compared to sterilized soil. The total level of phenolic compounds followed a similar pattern to that observed for chlorogenic acid, but only leaves of plants growing in pots inoculated with *F. ovina* (FO) soil

had higher concentrations of phenolic compounds than leaves of plants grown in sterilized soil (Table 1 and Fig. S6).

Note S3 Microbial richness and community composition of the chrysanthemum soils from the second experiment

From the second experiment, first we wanted to assess how the soil microbial composition and richness in inoculated soil in which chrysanthemum had been grown depended on the identity of the inoculum. Fungal and bacterial richness was as expected much higher in inoculated soils than in the sterilized one (Bacteria: $F_{1, 7}$=86.77, p<0.001; Fungi: $F_{1, 7}$=18.9, p=0.003; Fig. S10). When analyzing the effect of the specific soil inocula, all of them were different from sterilized soil for bacterial richness, and all but FO for fungal richness (Tukey; p>0.05). After excluding sterilized soil form the analysis, plant species conditioning the inocula or their functional group did not affect either the bacterial (plant species: $F_{7, 54}$=1.95, p=0.08; functional group: $F_{1, 6}$=0.81, p=0.40; Fig. S10a) or fungal (plant species: $F_{7, 42}$=1.82, p=0.11; functional group: $F_{1, 6}$=0.41, p=0.55; Fig. S10b) communities in chrysanthemum soil.

The bacterial and fungal community structure in chrysanthemum soil was strongly influenced by soil inoculation (Fig. S10). After excluding the sterilized soil from the analysis, plant species also affected the bacterial and fungal community structure (PERMANOVA; bacteria: F= 4.05, p<0.001; fungi: F=1.28, p<0.01; Fig. S10). Functional group in contrast, affected the community structure of bacteria (F= 2.45, p<0.005; Fig. S10c) but not of fungi (F= 1.29, p=0.11; Fig. S10d). The bacterial community in the soils that were inoculated with soils from LP and TM were the

most distinct from the rest, whereas in the fungal community soils containing RA inocula were the most distinct (Fig. S10).

After showing an effect of previous plant species on the bacterial and fungal community structure, we further explored which groups of microbes are affected by the soil conditioning and inoculation. The composition of the soils where chrysanthemum had grown (from the second experiment) in terms of bacteria phylum also differed between whether the soil was inoculated, the plant species conditioning the inocula and the functional group of these species. Out of the 26 bacterial and one archaeal phyla included in the analysis, the relative abundance of 17 bacterial phyla and of the only archaeal phylum in originally 100% sterilized soil significantly differed between the 100% sterilized soil and inoculated soils (Table S3, Fig. S10). Remarkably, although most phyla were more abundant in inoculated soils, there were relatively less Actinobacteria, Chlorobi and Chloroflexi in inoculated soils than in the sterilized soil (Table S3, Fig. S11). The relative abundance of 18 bacterial and the archaeal phyla was significantly affected by the plant species that were inoculated in the chrysanthemum soil (Table S3, Fig. 11). The strongest effect of plant species was seen for the bacterial phylum Gemmatimonadetes, which showed the lowest relative abundance in the soils inoculated with LP and TM inocula (Fig. S11). The functional group of the plant species that was used to conditioned the soil (grass or forb) significantly affected the relative abundance of 5 bacterial and the archaeal phyla: there were relatively more Berkelbacteria, Chlamydiae, Elusibacteria and Thaumarchaeota *(*Archaea*)* in soils inoculated with forb soil, and more Saccharibacteria and members of phylum TM6 in soils inoculated with grass soil (Table S3, Fig. S11).

Regarding the composition in terms of fungal phyla, out of the five phyla detected, only the relative abundance of Chytridiomycota differed with inoculation, being significantly higher in sterilized soil than in inoculated soils (Fig. S12; Table S3). Two of the five phyla (Ascomycota and Basidiomycota) significantly differed among inoculated soils with different plant species legacies, when the 100% sterilized soil treatment was excluded (Fig. S12; Table S3). Soil microbiomes with TM inocula showed the lowest relative abundance of these two phyla (Fig. S12). The functional group of the plant species that was used to conditioned the soil inocula (grass or forb) significantly affected the relative abundance of 2 fungal phyla (Table S3): there were relatively more unclassified Ascomycota and Mucoromycota in soils inoculated with grass than with forb legacies (Fig. S12).

At the fungal class level the relative abundance of Glomeromycotina, unclassified Ascomycota, Leotiomycetes, Sordariomycetes, Tremellomycetes was lower in sterilized soils than in inoculated soil while the relative abundance of Eurotiomycetes and Spizellomycetes was higher (Table S3). Plants species conditioning the inocula affected the abundance in the soil of only one class (Saccharomycetes). The relative abundance of three classes (Pezizomycetes Pucciniomycetes, Tremellomycetes) was significantly higher in soils containing grass- than forb-inocula (Table S3).

Figures S1-S12

Fig. S1 Richness of bacteria and fungal phylotypes in the inocula (upper panels) and in the soil after chrysanthemum growth (bottom panels). Data are from experiment 1 and the mean Chao1 index (± SE) is shown. Sterilized bulk soil was inoculated with 10% sterile soil (sterilized) or soil conditioned by grasses (AP: *Alopecurus pratensis,* FO: *Festuca ovina,* HL: *Holcus lanatus,* LP: *Lolium perenne*) or forbs (AM: *Achillea millefolium,* GM: *Galium mollugo,* RA: *Rumex acetosella,* TM: *Tripleurospermum maritimum*). “Soil” represents the effect of soil inocula, and here the sterilized soil is excluded from the analysis. When included, all inoculated soils were different from the sterilized soil for bacteria, and all but AP and FO for fungi. ***P < 0.001, *P < 0.05, *ns*: not significant.

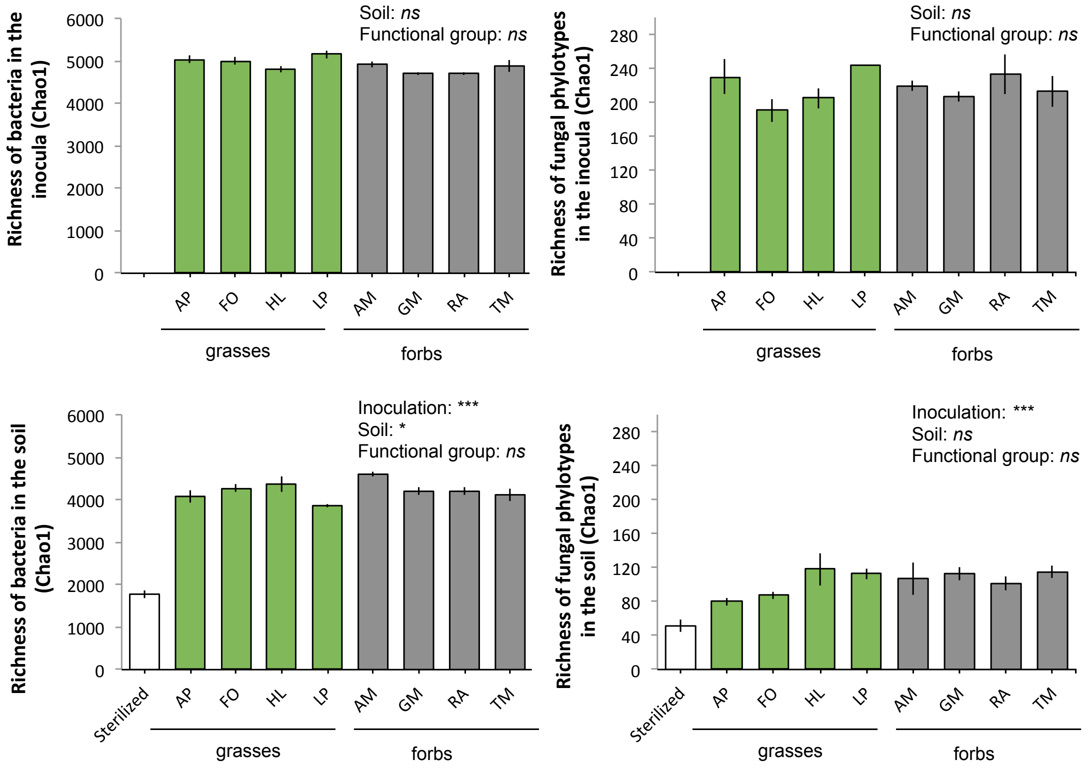

Fig. S2 Correlations between the richness (Chao1 index) of bacteria (left) and fungi (right) in the inocula and in the soils after chrysanthemum growth in experiment 1. Inocula were conditioned by either forbs (upper panels) or grasses (bottom panels).

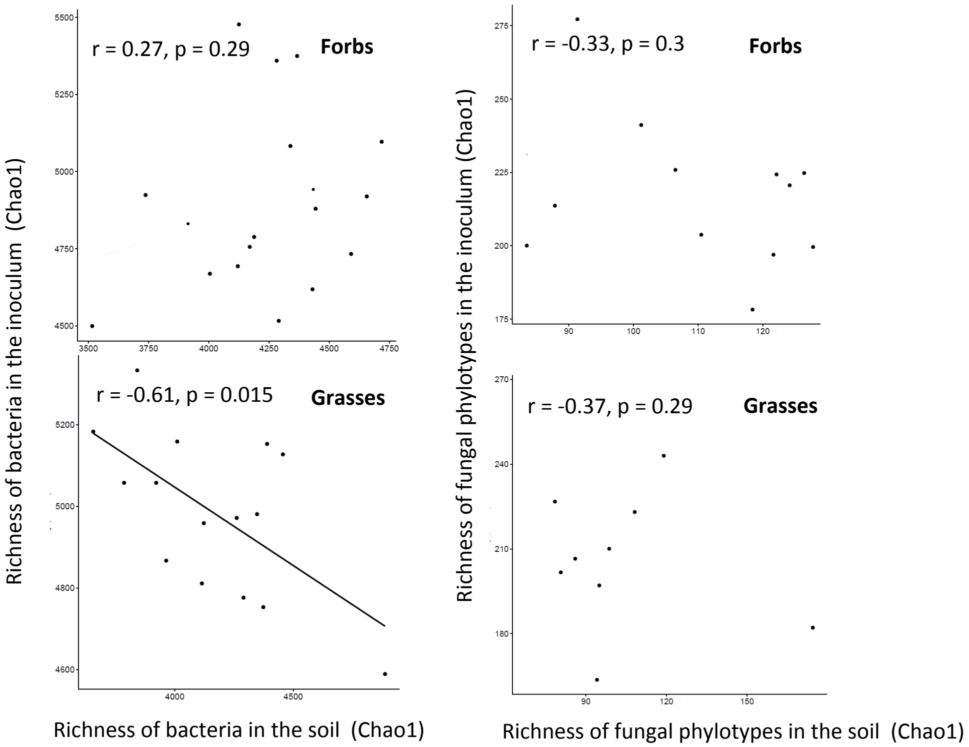

Fig. S3 Richness (Chao1) of fungal phylotypes of the subphylum Glomerycotina, commonly known as Arbuscular Mycorrhizal fungi. X-axis shows soil inocula conditioned by the different plant species and their corresponding inoculated chrysanthemum soil. Inocula were conditioned by grasses (AP: *Alopecurus pratensis,* FO: *Festuca ovina,* HL: *Holcus lanatus,* LP: *Lolium perenne*) or forbs (AM: *Achillea millefolium,* GM: *Galium mollugo,* RA: *Rumex acetosella,* TM: *Tripleurospermum maritimum*).Tukey box-and-whisker plots show median richness, 90 percentile quartiles and range while the dots depicts individual samples.

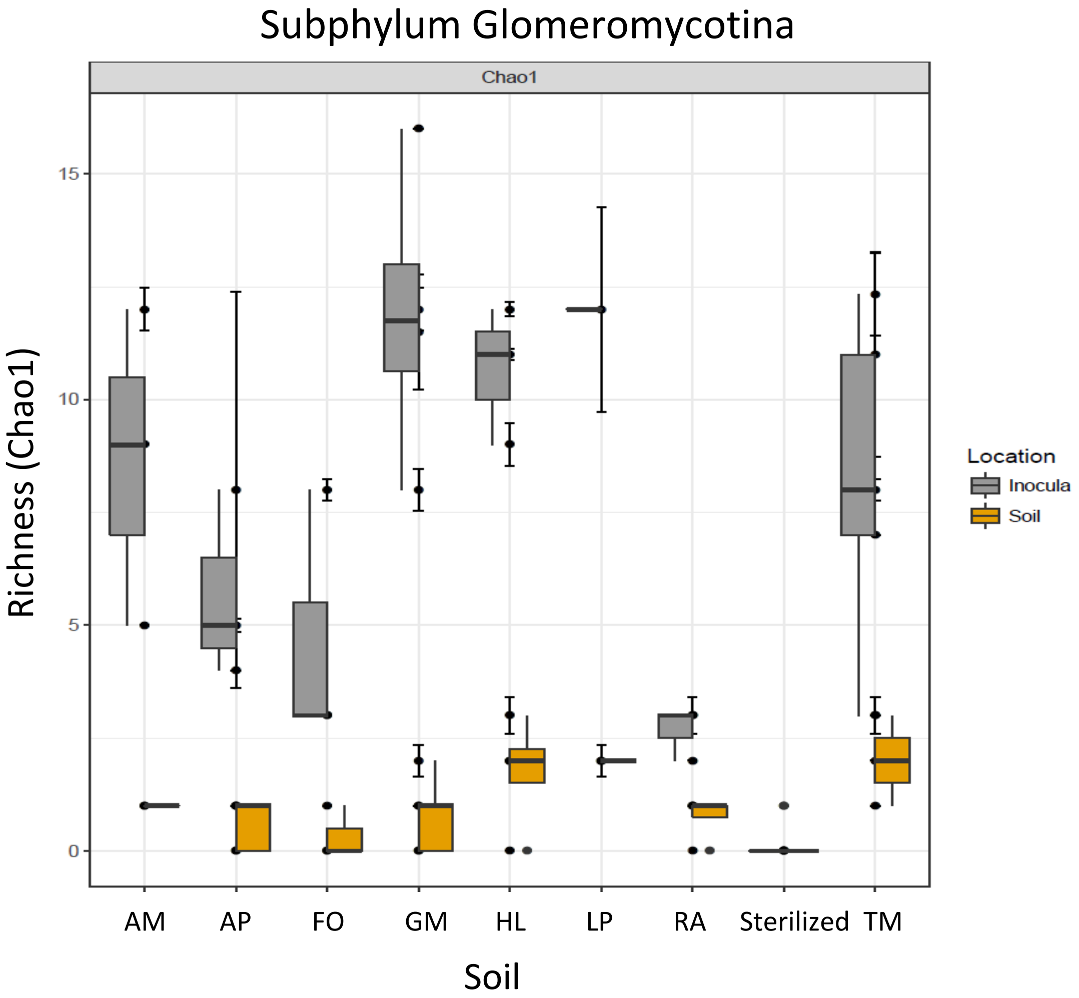

Fig. S4 Effects of soil inoculation on chrysanthemum biomass of plants from the first experiment. Sterilized soil was inoculated with 10% sterile soil or soil conditioned by grasses (AP: *Alopecurus pratensis,* FO: *Festuca ovina,* HL: *Holcus lanatus,* LP: *Lolium perenne*) or forbs (AM: *Achillea millefolium,* GM: *Galium mollugo,* RA: *Rumex acetosella,* TM: *Tripleurospermum maritimum*). Bars represent means ± SE (n=16 plants). These parameters are analyzed for the effect of overall inoculation (inoculated or sterilized), specific soil inocula (eight conditioned soils plus sterilized), and functional group (grasses or forbs, excluding sterilized); asterisks above bars indicate significant differences with the sterilized soil (Dunnett test). ***P < 0.001, *P < 0.05, *ns*: not significant.

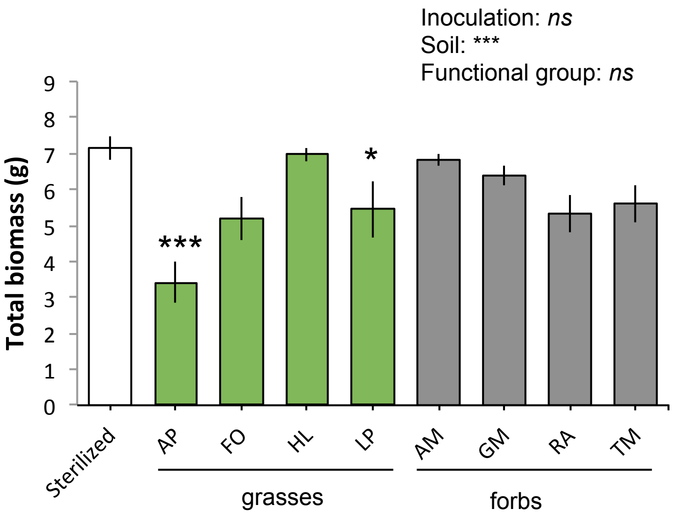

Fig. S5 Effects of soil inoculation on thrips survival, measured as number of emerged adults from a total of 5 initial thrips nymphs from the second experiment. Sterilized soil was inoculated with 10% sterile soil or soil conditioned by grasses (AP: *Alopecurus pratensis,* FO: *Festuca ovina,* HL: *Holcus lanatus,* LP: *Lolium perenne*) or forbs (AM: *Achillea millefolium,* GM: *Galium mollugo,* RA: *Rumex acetosella,* TM: *Tripleurospermum maritimum*). In this second experiment plants were grown in an enclosed greenhouse and herbivore performance was assessed in detached leaf assays. Bars represent means ± SE (n=10 plants; estimated means for generalized linear models). These parameters are analyzed for the effect of overall inoculation (inoculated or sterilized), specific soil inocula (eight conditioned soils plus sterilized), and functional group (grasses or forbs, excluding sterilized); *ns*: not significant.

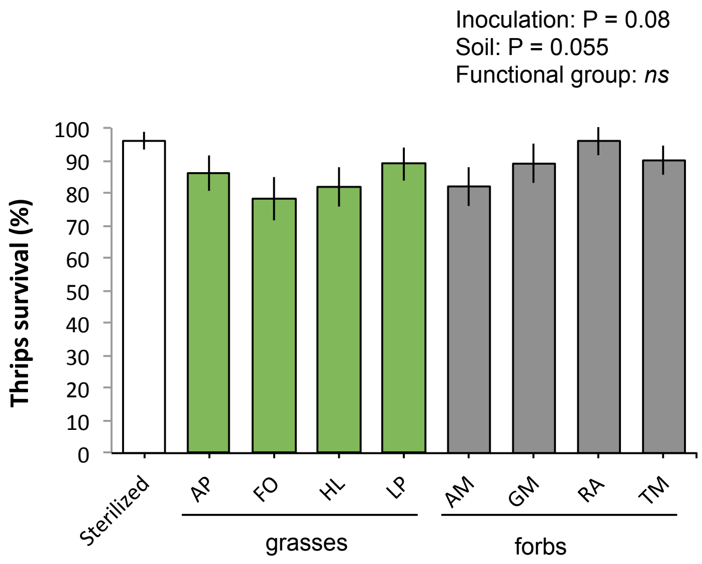

Fig. S6 Effects of soil inoculation on Phenolics in leaves from uninfested plants. Sterilized soil was inoculated with 10% sterile soil or soil conditioned by grasses (AP: *Alopecurus pratensis,* FO: *Festuca ovina,* HL: *Holcus lanatus,* LP: *Lolium perenne*) or forbs (AM: *Achillea millefolium,* GM: *Galium mollugo,* RA: *Rumex acetosella,* TM: *Tripleurospermum maritimum*). In this second experiment plants were grown in an enclosed greenhouse and herbivore performance was assessed in detached leaf assays. Bars represent means ± SE (n=10 plants). These parameters are analyzed for the effect of overall inoculation (inoculated or sterilized), specific soil inocula (eight conditioned soils plus sterilized), and functional group (grasses or forbs, excluding sterilized). Asterisks above bars indicate significant differences with the sterilized soil (Dunnett test); **P < 0.01, *ns*: not significant.

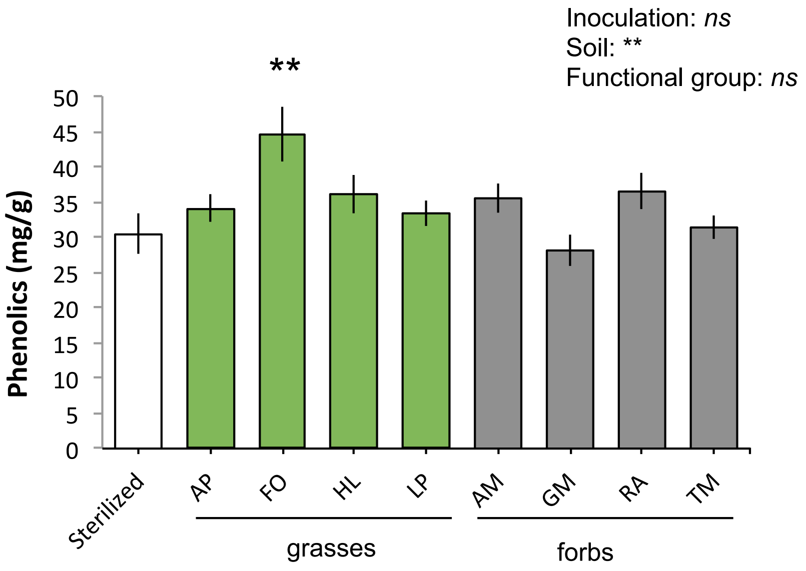

Fig. S7 The relative abundance of bacterial (a) and fungal (b) groups showing the strongest correlation with thrips performance (measured as the number of thrips reaching pupal stage) in the second experiment. Tukey box-and-whisker plots show median diversity, 90 percentile quartiles and range while the dots depicts individual samples.

a)

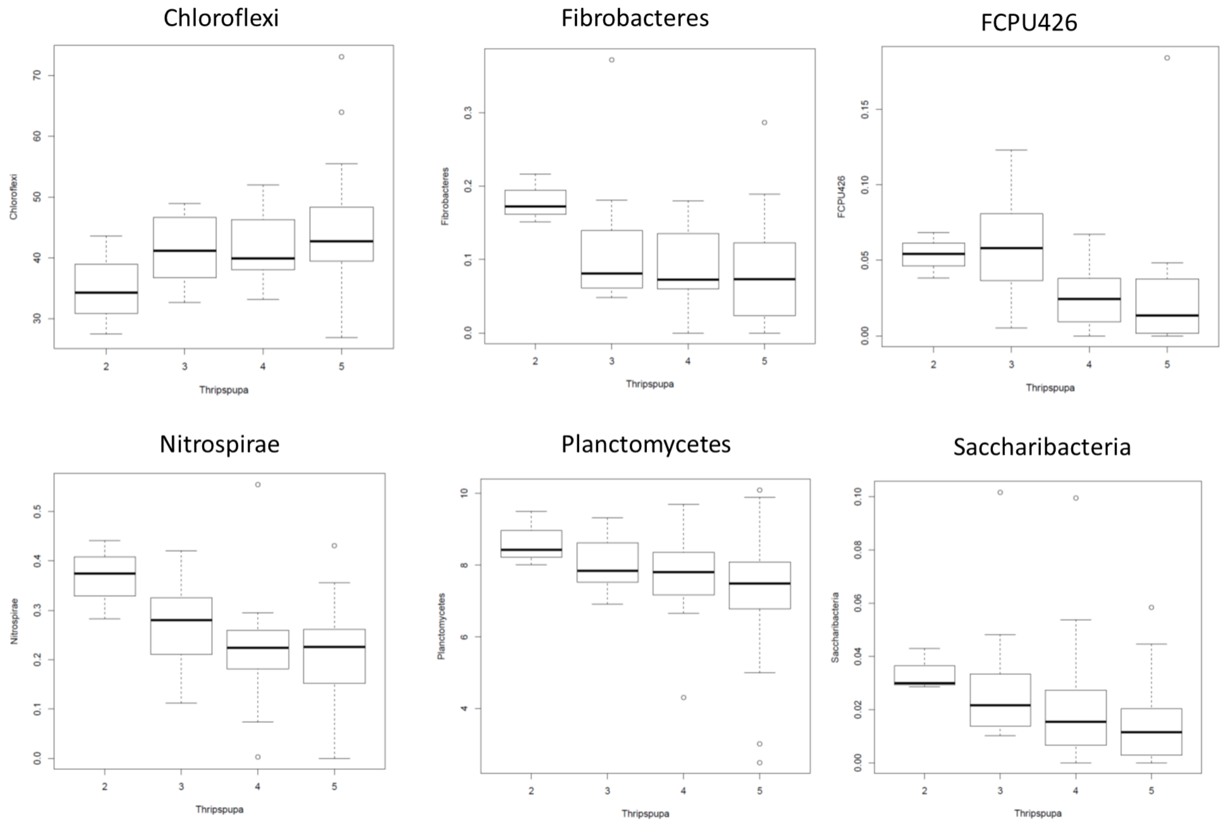

b)

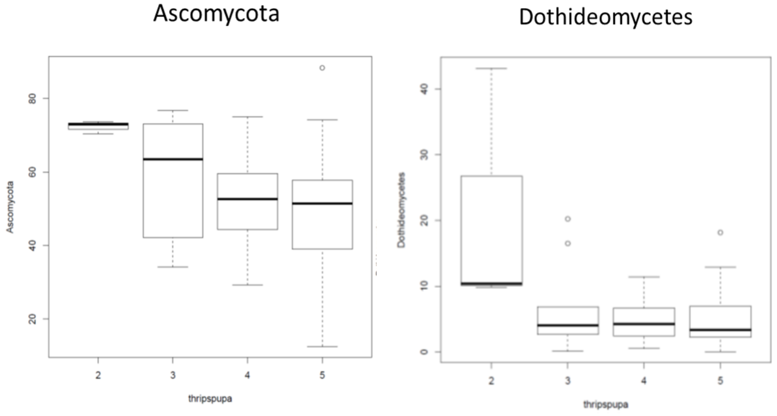

Fig. S8 Correlations between parameters of plant performance and resistance and relative abundance of fungal and bacterial taxa in the soil after chrysanthemum growth from the second experiment. The scale color of the filled squares indicates the strength of the linear Pearson correlation coefficients (r) and whether it is negative (red) or positive (blue). Only significant correlations with p<0.05 after Bonferroni correction are shown. If the correlation is not significant, the box is left white.

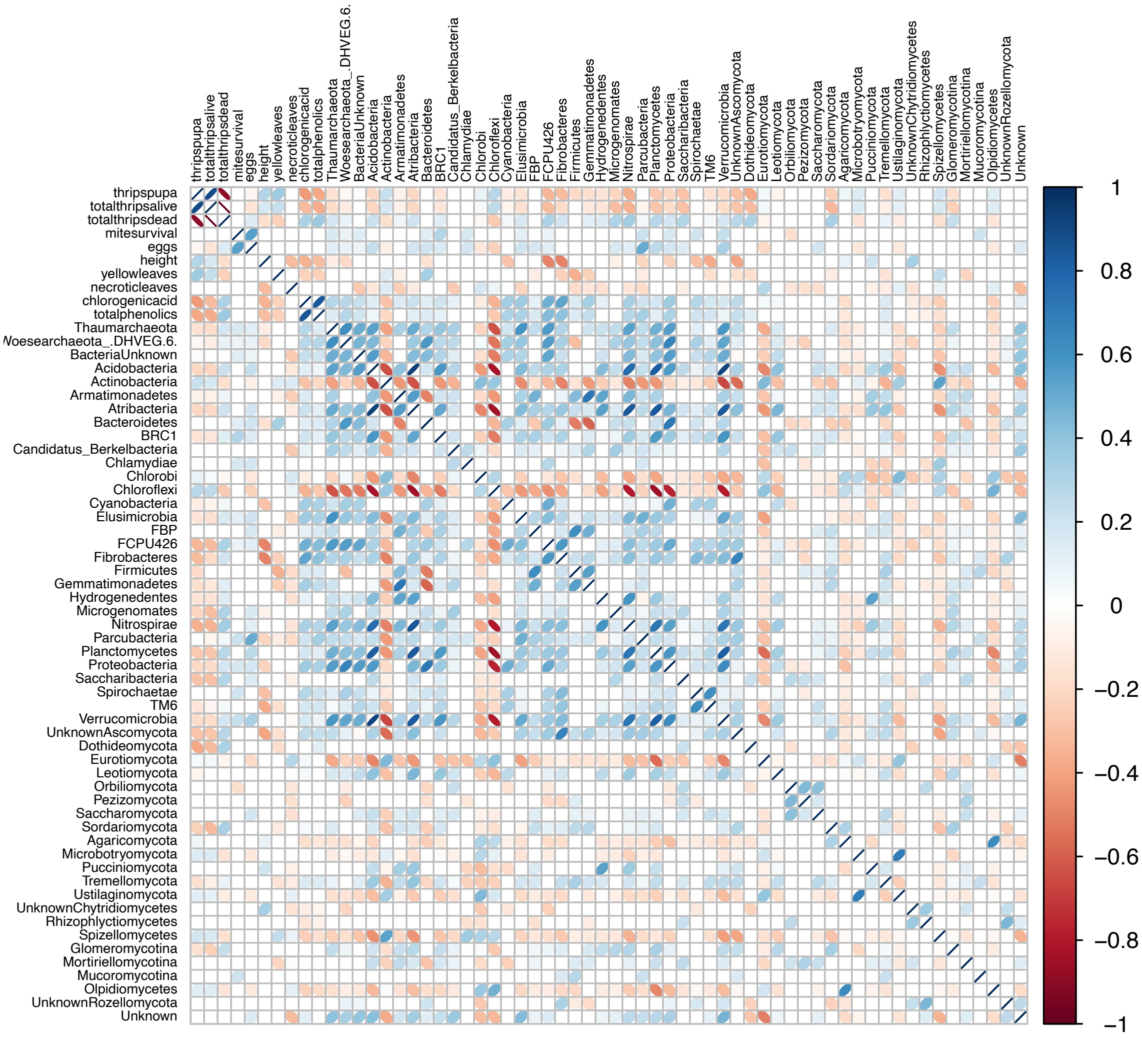

Fig. S9 Correlations at the closest level to genus between parameters of plant performance and resistance and relative abundance of fungal and bacterial taxa in the soil after chrysanthemum growth from the second experiment. The scale color of the filled squares indicates the strength of the linear Pearson correlation coefficients (r) and whether it is negative (red) or positive (blue). Only significant correlations with p<0.05 after Bonferroni correction are shown. If the correlation is not significant, the box is left white. For fungi, the functions are indicated between brackets: P= potential pathotroph, pP = potential plant pathogen and aP= potential animal pathogen, S=Saprotoph, M=mutualist, u=unknown.

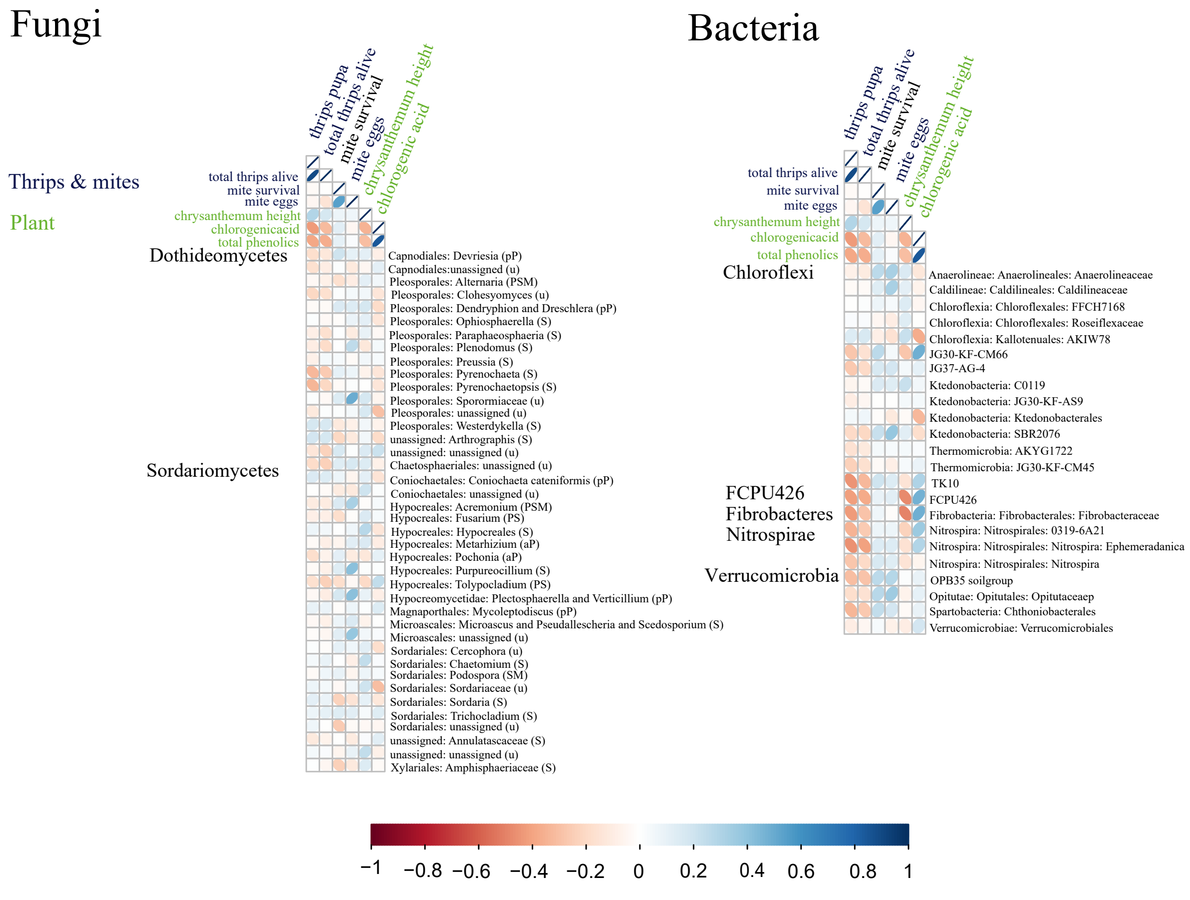

Fig. S10 Bacterial and fungal richness (top panels) and community composition (bottom panels) in the soil after chrysanthemum growth. Data are from soils of the second experiment. Sterilized soil was inoculated with 10% sterile soil or soil conditioned by grasses (AP: *Alopecurus pratensis,* FO: *Festuca ovina,* HL: *Holcus lanatus,* LP: *Lolium perenne*) or forbs (AM: *Achillea millefolium,* GM: *Galium mollugo,* RA: *Rumex acetosella,* TM: *Tripleurospermum maritimum*). Bars in a) and b) represent the mean Chao1 index (± SE, n=4-9 replicates). "Soil" represents the effect of soil inocula, and here the sterilized soil is excluded from the analysis. When included, all inoculated soils were different from the sterilized soil for bacteria, and all but FO for fungi. ***P < 0.001, *P < 0.05, *ns*: not significant. Centroids in c) and d) are shown as large dots and lines connect the individual samples to the centroids.

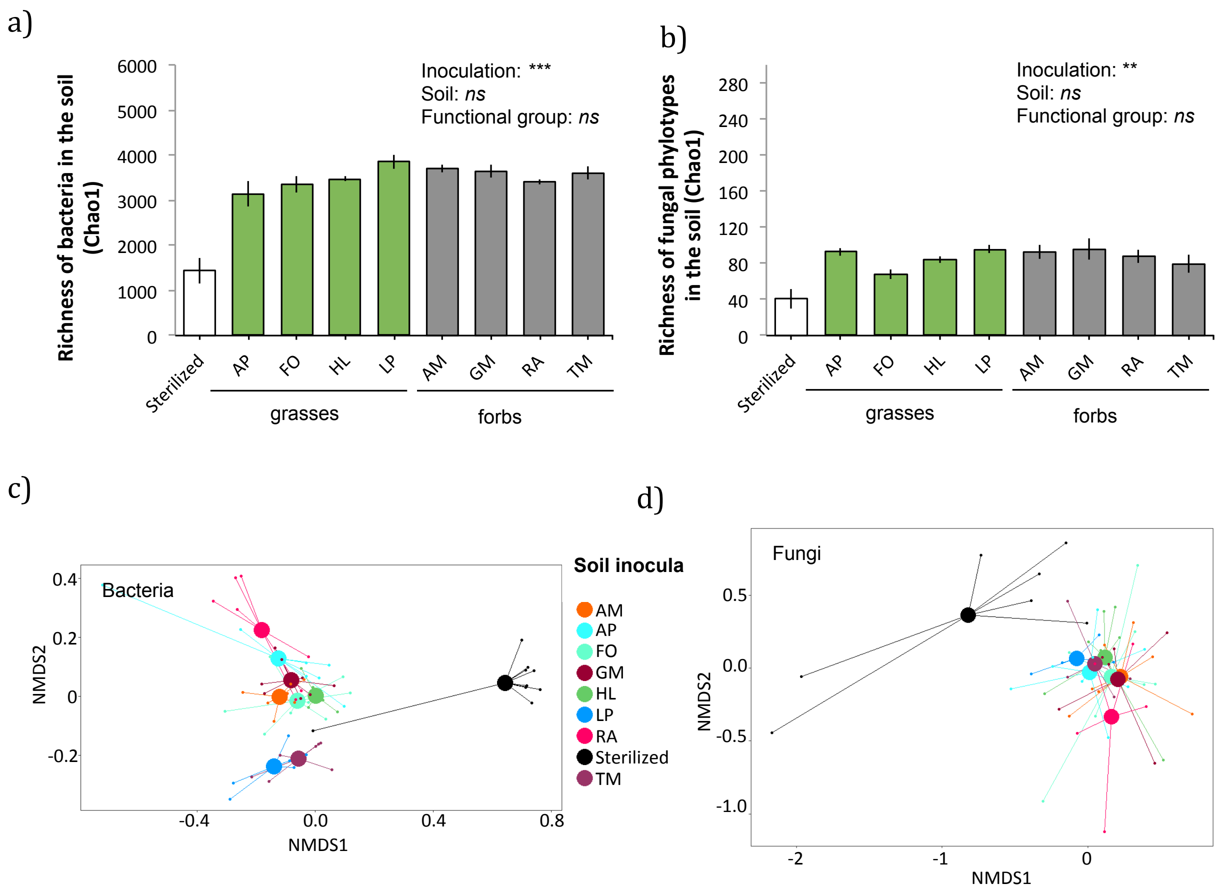

Fig. S11 The relative abundance of bacterial phyla that were affected by the soil conditioning and inoculation in the second experiment. Inocula were conditioned by grasses (AP: *Alopecurus pratensis,* FO: *Festuca ovina,* HL: *Holcus lanatus,* LP: *Lolium perenne*) or forbs (AM: *Achillea millefolium,* GM: *Galium mollugo,* RA: *Rumex acetosella,* TM: *Tripleurospermum maritimum*), or was sterilized soil (ST). Bars are depicted by soil inocula and ordered by functional group. Tukey box-and-whisker plots show median abundance, 90 percentile quartiles and range while the dots depicts individual samples.

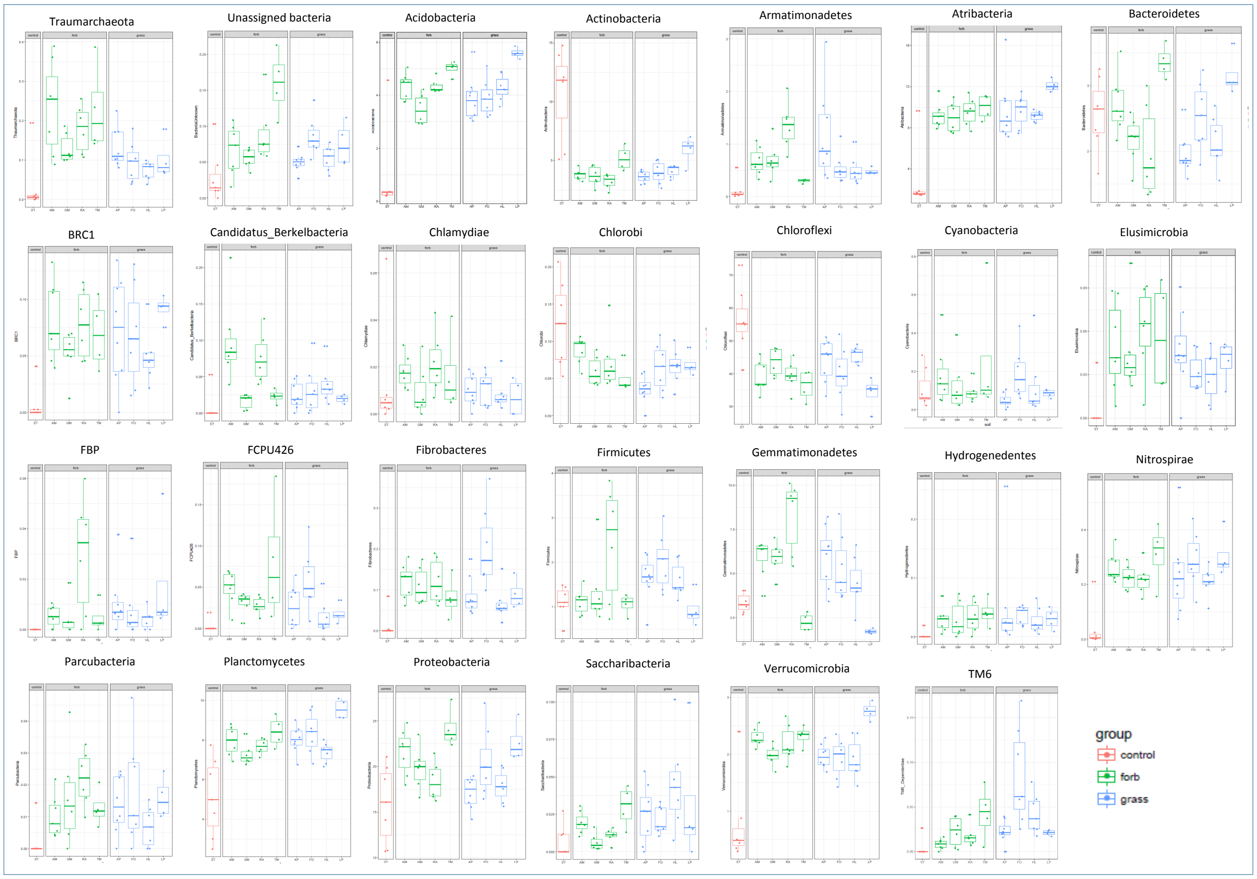

Fig. S12 The relative abundance of fungal phyla that were affected by the soil conditioning and inoculation in the second experiment. Inocula were conditioned by grasses (AP: *Alopecurus pratensis,* FO: *Festuca ovina,* HL: *Holcus lanatus,* LP: *Lolium perenne*) or forbs (AM: *Achillea millefolium,* GM: *Galium mollugo,* RA: *Rumex acetosella,* TM: *Tripleurospermum maritimum*), or was sterilized soil (ST).Bars are depicted by soil inocula and ordered by functional group. Tukey box-and-whisker plots show median abundance, 90 percentile quartiles and range while the dots depicts individual samples.

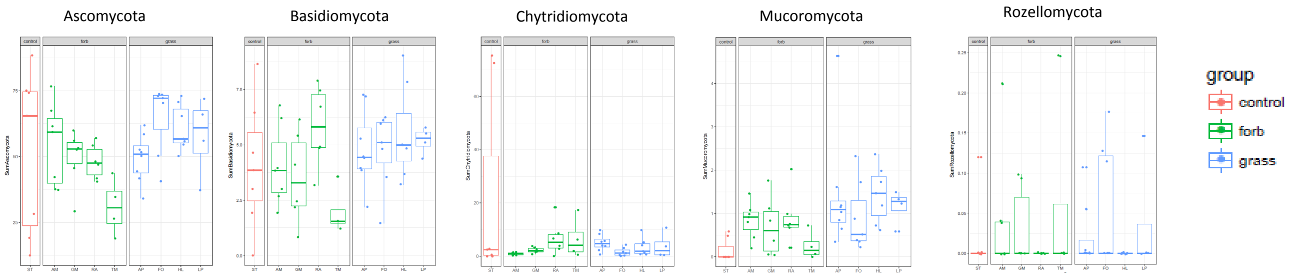

Supplement: Supplementary file 1 — Fig. S1 Richness of bacteria and fungal phylotypes in the inocula (upper panels) and in the soil after chrysanthemum growth (bottom panels). Fig. S2 Correlations between the richness (Chao1 index) of bacteria (left) and fungi (right) in the inocula and in the soils after chrysanthemum growth in Expt 1. Fig. S3 Richness (Chao1) of fungal phylotypes of the subphylum Glomeromycotina. Fig. S4 Effects of soil inoculation on chrysanthemum biomass of plants from Expt 1. Fig. S5 Effects of soil inoculation on thrips survival from Expt 2. Fig. S6 Effects of soil inoculation on Phenolics in leaves from uninfested plants from Expt 2. Fig. S7 The relative abundance of bacterial (a) and fungal (b) groups showing the strongest correlation with thrips performance in Expt 2. Fig. S8 Correlations between parameters of plant performance and resistance and relative abundance of fungal and bacterial taxa in Expt 2. Fig. S9 Correlations at the genus level between parameters of plant performance and resistance and relative abundance of fungal and bacterial taxa in Expt 2. Fig. S10 Bacterial and fungal richness (top panels) and community composition (bottom panels) in the soil after chrysanthemum growth from Expt 2. Fig. S11 The relative abundance of bacterial phyla that were affected by the soil conditioning and inoculation in Expt 2. Fig. S12 The relative abundance of fungal phyla that were affected by the soil conditioning and inoculation in Expt 2. Methods S1 Herbivore rearing. Methods S2 Soil preparation and plant growth. Methods S3 Chemical analysis of phenolics. Notes S1 Soil inoculation effects on microbial richness in inocula and soils. Notes S2 Soil inoculation effects on plant growth and plant defenses. Notes S3 Microbial richness and community composition of the chrysanthemum soils from Expt 2. [file NPH-226-595-s001.pdf]
